# Progenitor exhausted PD-1^+^ T cells are cellular targets of immune checkpoint inhibition in atherosclerosis

**DOI:** 10.1038/s44161-025-00713-2

**Published:** 2025-10-07

**Authors:** Megan Mulholland, Anthi Chalou, Samuel H. A. Andersson, Marie A. C. Depuydt, Yinda Yu, Shiying Lin, Klara Tallbäck, Astrid Ericsson, Gabriel Jakobsson, Jill de Mol, Dmytro Kryvokhyzha, Andrew H. Lichtman, Amanda C. Foks, Alexandru Schiopu, Harry Björkbacka, Bram Slütter, Anton Gisterå, Daniel Engelbertsen

**Affiliations:** 1 Department of Clinical Sciences, Malmö, Cardiovascular Research – Immune Regulation, Lund University, Malmö, Sweden; 2 Leiden Academic Centre for Drug Research, Division of Biotherapeutics, Leiden University, Leiden, The Netherlands; 3 Department of Medicine Solna, Center for Molecular Medicine, Karolinska University Hospital, Karolinska Institutet, Stockholm, Sweden; 4 Department of Translational Medicine, Cardiac Inflammation, Lund University, Malmö, Sweden; 5 Department of Clinical Sciences, Malmö, Lund University Diabetes Centre, Lund University, Malmö, Sweden; 6 Department of Pathology, Brigham and Women’s Hospital, Harvard Medical School, Boston, MA USA; 7 Department of Clinical Sciences, Malmö, Cardiovascular Research – Cellular Metabolism and Inflammation, Lund University, Malmö, Sweden

**Keywords:** Immunotherapy, Cardiovascular biology, Adaptive immunity

## Abstract

Immune checkpoint inhibitors (ICIs), targeting checkpoint receptors such as programmed cell death protein 1 (PD-1), are associated with increased risk of cardiovascular events, but the underlying mechanisms remain poorly understood. Here we show that PD-1^+^ T cells from murine atherosclerotic aortas mainly display a progenitor exhausted phenotype (PD-1^int^Slamf6^+^Tim3^−^), produce IFNγ in vivo, exhibit signs of recent proliferation and maintain polyfunctionality. PD-1 blockade induced marked changes in plaque immune phenotype, with increased PD-1^high^ T cell accumulation, IFNγ production, formation of lymphocyte foci and neutrophil recruitment. Depletion of PD-1^high^ T cells prior to PD-1 blockade did not impede T cell recruitment, suggesting a role for progenitor exhausted PD-1^int^ T cells in ICI-driven T cell plaque accumulation. Human circulating PD-1^+^ T cells produced IFNγ and were associated with subclinical coronary atherosclerosis. Our studies highlight IFNγ-producing PD-1^+^ T cells as a potential key immune cell population mediating increased cardiovascular risk in patients with cancer receiving ICI.

## Abstract (web summary)

Mulholland et al. identify progenitor exhausted T cells, expressing intermediate levels of PD-1 (PD-1^int^), as a prominent source of pro-inflammatory cytokines in the murine atherosclerotic aorta and potential cellular targets driving checkpoint inhibition-elicited pro-atherosclerotic immune responses. They further demonstrate elevated levels of circulating PD-1-expressing T cells in individuals with subclinical cardiovascular disease.

## Main

Atherosclerosis is a non-resolving inflammatory disease in which T cells play a central role in modulating plaque inflammation by cytokine release (for example, IFNγ) and cell–cell interactions^1–3^. Loss of T cell inhibitory checkpoint receptor signaling promotes plaque inflammation and accumulation of T cells^4–7^. ICI therapies targeting PD-1 or its ligand, programmed death ligand 1 (PD-L1), have recently been shown to be associated with increased cardiovascular risk in patients with cancer^8–10^, but mechanistic insight into how checkpoint receptor inhibition translates to cardiovascular risk is lacking.

Immune checkpoint receptors are expressed on a wide range of T cells, including recently activated T cells, semi-exhausted T cells (retaining functional capacity, referred to as ‘progenitor exhausted’ or ‘stem-like’ T cells) and terminally exhausted T cells^11^. Repeated T cell receptor (TCR) signaling, caused by recurrent interactions between the TCR and cognate peptide presented on major histocompatibility complex class I (MHC-I) or MHC-II, drives persistent expression of T cell inhibitory immune checkpoint receptors, such as PD-1 and lymphocyte activation gene 3 (LAG3)^12^. Several studies have identified plaque T cells that express genes associated with T cell exhaustion^13–15^, but little is known regarding the function of these T cells in vivo and how they respond to ICI therapy.

In the present study, we characterized the functional characteristics of T cells expressing PD-1 in atherosclerotic plaques and identified PD-1^int^Slamf6^+^ progenitor exhausted T cells as the main T cell source of IFNγ in the murine atherosclerotic aorta. Treatment with PD-1 blocking antibodies promoted IFNγ secretion of aortic T cells and accumulation of T cells and neutrophils in plaques. Antibody-mediated depletion of PD-1^high^ T cells, sparing PD-1^int^ T cells, did not impede anti-PD-1-driven recruitment of T cells to plaques. Finally, suggesting a pro-atherosclerotic role in humans, PD-1^+^ T cells were enriched for IFNγ-producing cells, and levels of circulating PD-1^+^ T cells were associated with subclinical coronary atherosclerosis.

## Results

### IFNγ^+^ aortic T cells in hypercholesteremic mice are PD-1^+^

**Fig. 1:**
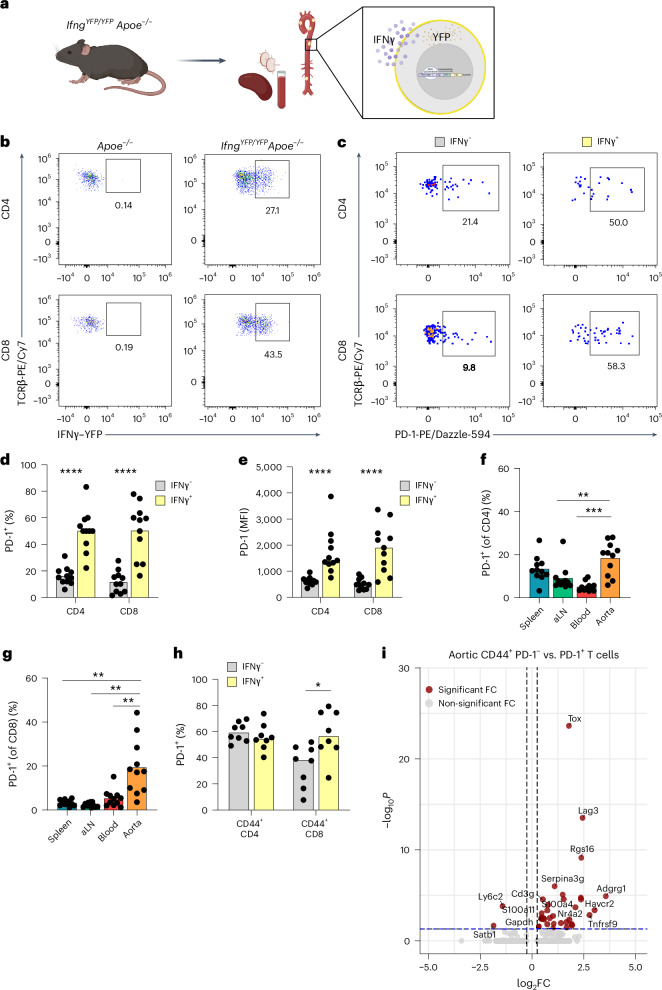
The majority of aortic IFNγ^+^ T cells in hypercholesteremic mice are PD-1^+^. **a**,**b**, IFNγ–YFP reporter mice were crossed onto an atherosclerotic background (*Apoe*^−*/*−^) and fed an HCD for 12 weeks, and flow cytometry was performed to characterize IFNγ-producing T cells (*n* = 11). **c**–**e**, Comparison of PD-1 expression between IFNγ–YFP^−^ and IFNγ–YFP^+^ aortic CD4 (****P = 1.8 × 10^−6^, 1.2 × 10^−5^) and CD8 T cells (****P = 5.7 × 10^−6^, 9.9 × 10^−5^). **f**,**g**, Comparison of PD-1 expression on CD4 (**P = 0.0068, ***P = 0.0002) and CD8 (**P = 0.0045, 0.0021, 0.0039, respectively) T cells between spleen, aortic-draining iliac lymph node (aLN), blood and aorta. **h**, PD-1 expression comparing antigen-experienced CD44^+^ IFNγ–YFP^+/−^ T cells. **i**, scRNA-seq analysis of differential gene expression comparing aortic antigen-experienced (CD44-expressing) T cells with or without PD-1 expression. **d**, Bars denote mean, analyzed with two-sided unpaired *t*-test. **e**, Bars denote median, analyzed with two-sided Mann–Whitney *U-*test. **f**,**g**, Bars denote mean, analyzed with two-sided repeated-measures one-way ANOVA test compared to aorta. **i**, Adjusted *P*value after FDR adjustment. *P* values are reported top-to-bottom and left-to-right. FC, fold change. Source Data

**Extended Data Fig. 1:**
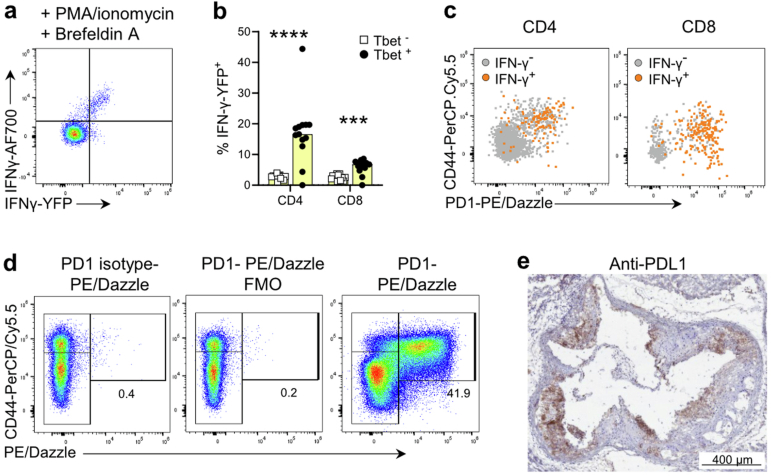
IFN-γ expression in PD1^+^ T cells. (**a**) Splenocytes from *Ifng*^*YFP/YFP*^*Apoe*^−*/*−^ mice were stimulated with PMA/ionomycin with Brefeldin A for 4 hr and analyzed for IFN-γ-YFP and IFN-γ-AF700 expression. (**b-d**) *Ifng*^*YFP/YFP*^*Apoe*^−*/*−^ mice were fed a high-cholesterol diet for 12 weeks, and flow cytometry was performed to characterize IFN-γ-producing T cells (n = 11). (**b**) Increased frequency of IFN-γ-YFP expression in Tbet^+^ splenic CD4 (****p = 6.9E-5) and CD8 T cells (***p = 0.0004) compared to Tbet^−^ T cells. (**c**) Overlay flow cytometry plots of IFN-γ^+^ (orange) and IFN-γ^−^ (grey) aortic CD4 and CD8 T cells. (**d**) Flow cytometry plots of PD1-PE/Dazzle antibody staining and matched controls, PE/Dazzle-rat IgG2a isotype control (PD1 isotype) and PD1-PE/Dazzle fluorescent minus-one (FMO), of splenic CD4 T cells. (**e**) Representative immunohistochemical staining of PDL1 in aortic root cross-section of *Ifng*^*YFP/YFP*^*Apoe*^−*/*−^ mice fed a high-cholesterol diet for 16 weeks (n = 4). (**b**) Bars denote median, analyzed with two-sided Mann-Whitney *U* test. Source Data

To characterize IFNγ-producing T cells in the atherosclerotic aorta, we generated hypercholesterolemic *Ifng*^*YFP/YFP*^*Apoe*^−*/*−^ mice. This cytokine reporter strain allows for detection of cells currently producing IFNγ, without the need for in vitro restimulation (Fig. 1a). Demonstrating specificity, yellow fluorescent protein (YFP) signal was present only in *Ifng*^*YFP/YFP*^*Apoe*^−*/*−^ mice (Fig. 1b) and was mainly restricted to Tbet^+^ CD4 and CD8 T cells, and YFP^+^ T cells co-localized with intracellular IFNγ protein using monoclonal anti-IFNγ antibodies (Extended Data Fig. 1a,b).

First, we evaluated the phenotype of aortic IFNγ^+^ T cells of *Ifng*^*YFP/YFP*^*Apoe*^−*/*−^ mice fed a high-cholesterol diet (HCD) for 12 weeks. Comparing IFNγ^+^ to IFNγ^−^ aortic T cells, we observed increased levels of PD-1 expression on IFNγ^+^ CD4 and CD8 T cells (Fig. 1c,d; CD4 *P* = 1.8 × 10^−6^, CD8 *P* = 1.2 × 10^−5^). Likewise, we observed increased levels of PD-1 mean fluorescence intensity (MFI) on both aortic IFNγ^+^ CD4 (*P* = 5.7 × 10^−6^) and IFNγ^+^ CD8 (*P* = 9.9 × 10^−5^) T cells when compared to IFNγ^−^ T cells (Fig. 1e and Extended Data Fig. 1c,d). Evaluating levels of PD-1^+^ T cells in various tissues, we observed higher levels of aortic PD-1^+^ CD4 T cells relative to lymph nodes (*P* = 0.0068) and blood (*P* = 0.0002), whereas aortic PD-1^+^ CD8 T cells were increased relative to spleen (*P* = 0.0045), lymph nodes (*P* = 0.0021) and blood (*P* = 0.0039) (Fig. 1f,g). Restricting our analysis to antigen-experienced T cells (CD44^+^), we found that IFNγ positivity in CD8 T cells was associated with PD-1 expression, but no such association was observed comparing antigen-experienced CD4 T cells (Fig. 1h). To further study the phenotype of PD-1-expressing T cells in the aorta, we analyzed single-cell RNA sequencing (scRNA-seq) data of aortic cells in low-density lipoprotein (LDL)-deficient mice^16^. Comparing gene expression between PD-1^+^ and PD-1^−^ memory T cells, we observed expression of several transcripts associated with T cell exhaustion and effector function, including *Tox*, *Rgs16*, *Lag3* and *Havcr2* (Fig. 1i and Supplementary Table 1). We confirmed the presence of the main ligand for PD-1, PD-L1, in plaques, mainly restricted to macrophage-rich areas (Extended Data Fig. 1e), as was previously reported^17^.

### PD-1^+^ aortic T cells display a progenitor exhausted phenotype

**Fig. 2:**
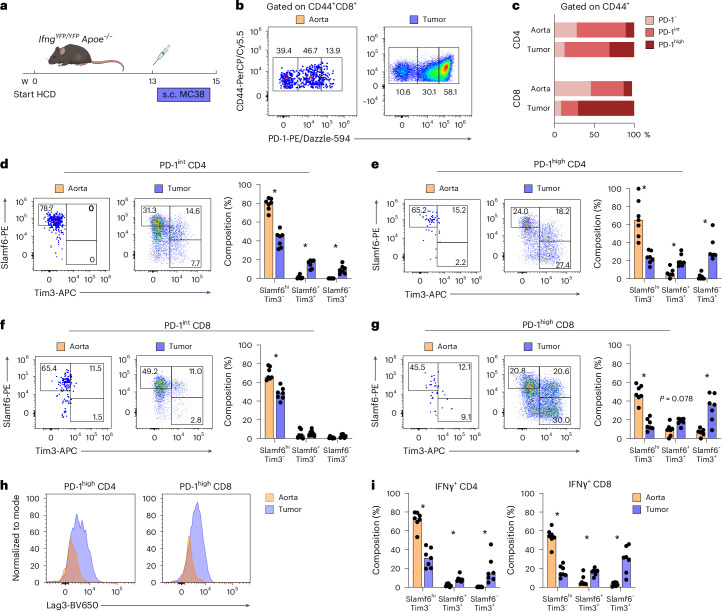
PD-1^+^ aortic T cells display a progenitor exhausted Slamf6^hi^Tim3^−^ phenotype. **a**, *Ifng*^*YFP/YFP*^*Apoe*^−*/*−^ mice (*n* = 7) were fed an HCD and injected subcutaneously with MC38 tumor cells. Aortas and tumors were harvested 2 weeks after tumor implantation and a total of 15 weeks on diet. **b**, Antigen-experienced CD44^+^ T cells were stratified based on the degree of cell surface PD-1 expression: CD44^+^PD-1^−^ (PD-1^−^), CD44^+^PD-1^intermediate^ (PD-1^int^) and CD44^+^PD-1^high^ (PD-1^high^). **c**, PD-1 subset composition of T cells in the aorta and tumor. **d**–**g**, Flow cytometry plots and quantification of stratification of T cells into three subsets of PD-1^+^ T cells based on expression of Slamf6 and Tim3 (*P = 0.016; CD8 PD-1^high^ Slamf6^−^Tim3^+^ *P = 0.031). **h**, Expression of Lag3 on PD-1^high^ CD4 and CD8 T cells from aorta and tumor. **i**, Expression of Slamf6 and Tim3 on IFNγ-producing CD4 and CD8 T cells in aorta and tumor (*P = 0.016; IFNγ^+^ CD8 Slamf6^+^Tim3^+^ *P = 0.031). **d**–**g**,**i**, Bars denote median, analyzed with two-sided Wilcoxon matched-pairs signed-rank test. s.c., subcutaneous; w, week. Source Data

**Extended Data Fig. 2:**
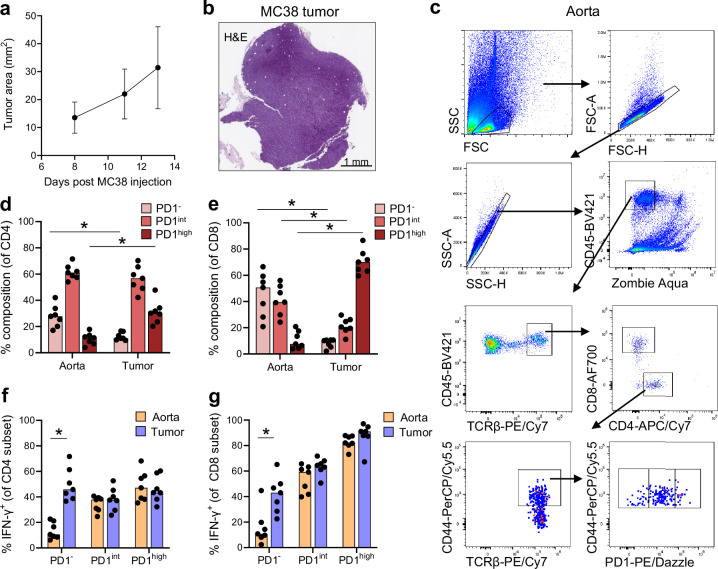
Comparative analysis of aortic and tumor-infiltrating exhausted T cells. *Ifng*^*YFP/YFP*^*Apoe*^−*/*−^ mice (n = 7) were fed a high-cholesterol diet and injected with MC38 tumor cells. Aortas and tumors were harvested 2 weeks after tumor implantation. T cells were stratified based on the degree of cell-surface PD1 expression: CD44^+^PD1^−^ (PD1^−^), CD44^+^PD1^intermediate^ (PD1^int^), and CD44^+^PD1^high^ (PD1^high^). (**a**) Tumor growth over time. Data presented as mean values +/- SD. (**b**) H&E staining of MC38 tumor (d14). (**c**) Flow cytometry gating strategy for identifying T cell PD1 subsets. (**d-e**) Frequency of PD1 CD4 and CD8 subsets of T cells in the aorta and tumor. (**f-g**) IFN-γ production by each PD1 T cell subset in the aorta and tumor. (**d-g**) Bars denote median, analyzed with two-sided Wilcoxon matched-pairs test (*p = 0.016). Source Data

Upon repeated antigen encounter, T cells may lose proliferative and functional capacity, upregulate inhibitory immune checkpoint receptors (PD-1 and LAG3) and develop through a progenitor exhausted state to a terminally exhausted state^11,12^. These subsets have been extensively characterized in tumor-infiltrating T cells^11,12,18^, but understanding of plaque PD-1^+^ T cells is lacking. To gain a better perspective of the phenotype of PD-1^+^ T cells in the atherosclerotic aortas, we designed a study to compare the phenotype of aortic T cells with tumor-infiltrating T cells. We implanted MC38 colon adenocarcinoma cells into the flank of HCD-fed *Ifng*^*YFP/YFP*^*Apoe*^−*/*−^ mice (*n* = 7) and harvested aortas and tumors 2 weeks after inoculation (Fig. 2a and Extended Data Fig. 2a,b).

We observed graded PD-1 expression (PD-1^int^ and PD-1^high^) in both aortas and tumors, with higher percentages of PD-1^high^ CD4 and CD8 T cells in the tumor compared to the aorta (Fig. 2b,c and Extended Data Fig. 2c–e). We then analyzed T cells based on expression of the surface proteins Slamf6 and Tim3, which were previously shown to stratify T cells into different exhaustion states^18^. We outlined three subsets of PD-1^+^ T cells: Slamf6^+^Tim3^−^ (progenitor exhausted), Slamf6^+^Tim3^+^ (early exhaustion) and Slamf6^−^Tim3^+^ (late exhaustion). We observed a pattern of increased levels of late exhausted Tim3-expressing PD-1^+^ T cells in tumors compared to aortas (Fig. 2d–g). Notably, aortic PD-1^int^ and PD-1^high^ CD4 and CD8 T cells were almost exclusively of a Slamf6^+^Tim3^−^ progenitor exhausted phenotype. Of note, the aortic T cell population that displayed the highest fraction of ‘late exhausted’ cells was PD-1^high^ CD8 T cells where approximately 5–10% were Slamf6^−^Tim3^+^ (Fig. 2g). The inhibitory checkpoint receptor Lag3 is co-expressed along with PD-1 on exhausted T cells in tumors^12^. Consistent with the elevated state of exhaustion in tumors compared to the aorta, we found a higher level of Lag3 expression on tumor PD-1^high^ CD4 and CD8 T cells compared to aorta (Fig. 2h).

In the atherosclerotic aorta, IFNγ–YFP^+^ CD4 and CD8 T cells were predominantly Slamf6^+^Tim3^−^ progenitor exhausted T cells, whereas cytokine-producing T cells in the tumor had a mixed phenotype (Fig. 2i). This was not due to differential capacity for IFNγ production of PD-1^int^ and PD-1^high^ T cells in the aorta compared to tumor (Extended Data Fig. 2f,g) but, rather, due to increased abundance of progenitor exhausted T cells in the atherosclerotic aorta (Fig. 2d–g). Altogether, these findings demonstrate that PD-1 expression in atherosclerotic aortas denotes cytokine-producing T cells with a progenitor exhausted phenotype, distinct from exhausted T cells found in tumors.

### Aortic PD-1-expressing T cells are polyfunctional

**Fig. 3:**
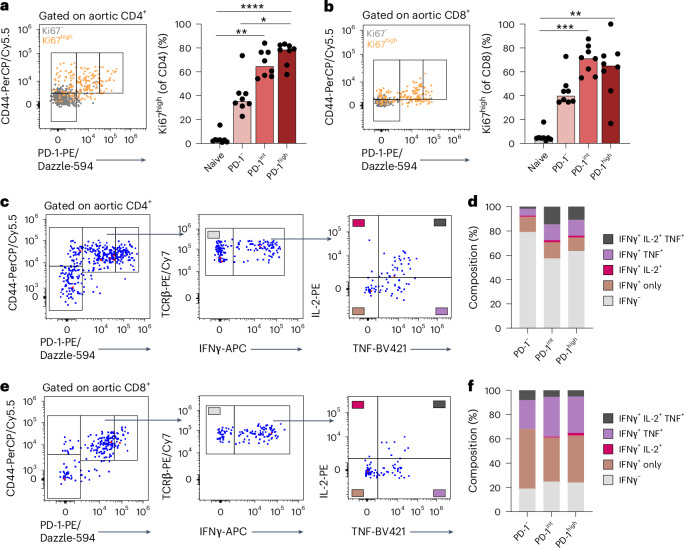
Aortic PD-1-expressing T cells are polyfunctional. **a**,**b**, Aortas from *Ifng*^*YFP/YFP*^*Apoe*^−*/*−^ mice were digested and pooled (*n* = 2 per pool, eight pools total). Representative flow cytometry and quantification of high Ki67 expression within CD44^+^PD-1 subsets of aortic CD4 (****P = 1.9 × 10^−5^, **P = 0.0014, *P = 0.033) and CD8 (***P = 0.0001, **P = 0.0013) T cells. **c**–**f**, Digested and pooled aortas (*n* = 3–4 per pool, three pools total) were stimulated ex vivo with PMA/ionomycin/brefeldin A for 4 hours, followed by flow cytometry. Each PD-1 subset within CD4 and CD8 T cells was analyzed for cytokine production and co-production of IFNγ (measured here with anti-IFNγ-APC antibody), TNF and IL-2. **a**,**b**, Bars denote median, analyzed with two-sided Kruskal–Wallis test. Source Data

**Extended Data Fig. 3:**
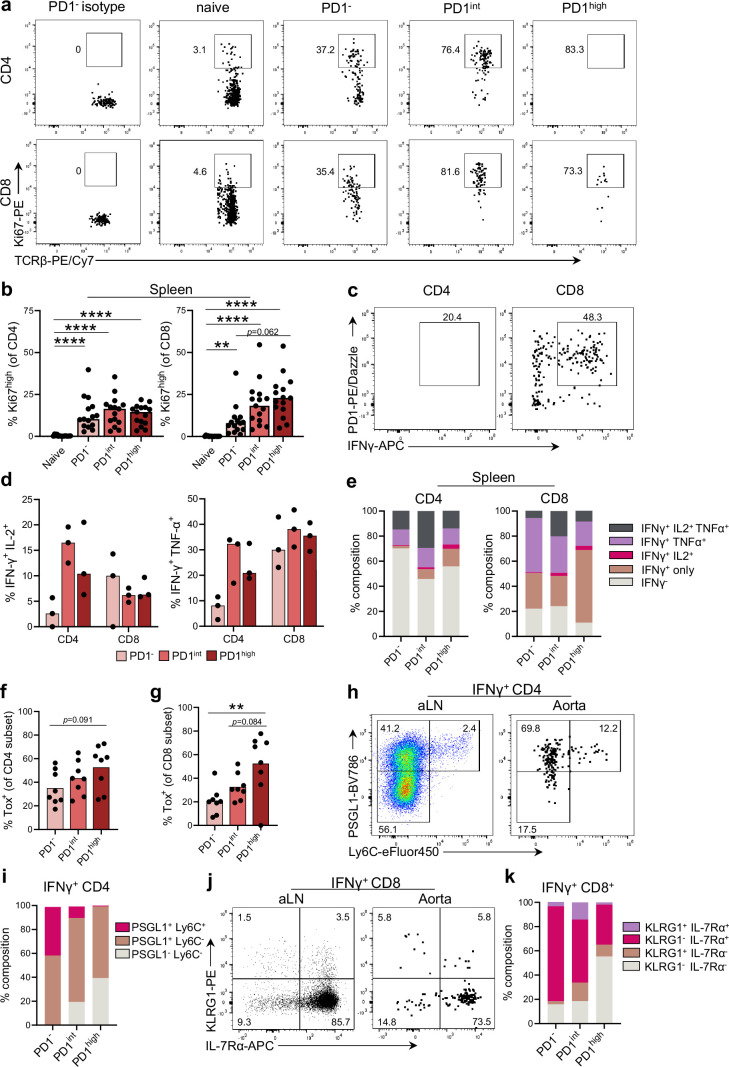
Functional characteristics of PD1-expressing aortic T cells. *Ifng*^*YFP/YFP*^*Apoe*^−*/*−^ mice were fed a high-cholesterol diet (12-16 weeks per experiment) and Ki67 expression and T-cell cytokine co-produciton was analyzed. (**a-b**) Digested whole-aortas were pooled (n = 2/pool; 8 pools total) and analyzed for proliferation along with splenocytes (n = 16). (**a**) Representative flow cytometry plots of Ki67-staining in naïve (CD44^−^) and PD1 subsets of aortic CD4 and CD8 aortic T cells. (**b**) Quantification of high Ki67 expression within PD1 subsets of splenic CD4 (top-down, ****p = 2.3E-5, 3E-6, 4.8E-5) and CD8 T cells (****p = 4E-8, 2E-6, **p = 0.0074). (**c-e**) Digested and pooled whole-aortas (n = 3-4/pool, 3 pools total) and spleens (n = 11) were stimulated *ex vivo* with PMA/ionomycin with Brefeldin A for 4 hr followed by flow cytometry. (**c**) Flow cytometry plot of enrichment of IFN-γ-APC expression in stimulated PD1^+^ aortic CD4 and CD8 T cells. (**d**) Frequency of co-production of IFN-γ (measured here with anti-IFN-γ-APC antibody) and IL-2 or IFN-γ and TNF-α by stimulated aortic CD4 and CD8 PD1 subsets. (**e**) Analysis of cytokine production and co-production of IFN-γ (measured here with anti-IFN-γ-APC antibody), TNF-α, and IL-2 with stimulated splenic PD1 CD4 and CD8 T cell subsets. (**f-g**) Frequency of Tox expression within aortic CD4 PD1 subsets and CD8 PD1 subsets (**p = 0.0046). (**b**) Bars denote median, analyzed with two-sided Kruskal Wallis test. (**h-i**) Quantification of PSGL1 and Ly6C on aortic IFN-γ^+^ CD4 T cell PD1-subsets. (**j**-**k**) Quantification of KLRG1 and IL7Rα on aortic IFN-γ^+^ CD8 T cell PD1-subsets. Representative flow cytometry plots of aorta-draining lymph node and aorta. (**f-g**) Bars denote mean, analyzed with two-sided one-way ANOVA test. Source Data

Exhausted T cells display diminished functional capacity, whereas progenitor exhausted T cells often display a high degree of polyfunctionality^12^. Indicative of recent proliferation, Ki67 expression in PD-1^int^ and PD-1^high^ subsets was not reduced compared to CD44^+^PD-1^−^ counterparts (Fig. 3a,b and Extended Data Fig. 3a). Likewise, we did not observe any drop in Ki67 levels among PD-1-expressing T cells in the spleen compared to PD-1^−^ T cells (Extended Data Fig. 3b). Next, we tested the capacity of PD-1^+^ aortic T cells to co-produce interleukin 2 (IL-2) and TNF along with IFNγ, as loss of cytokine ‘polyfunctionality’ is associated with terminal exhaustion^18^. To this end, we stimulated pooled digested whole aortas ex vivo (*n* = 3–4 aortas per pool, three pools total) with phorbol myristate acetate (PMA)/ionomycin/brefeldin A and analyzed T cell cytokine production capacity by flow cytometry. We validated that IFNγ production in the aorta, here measured by anti-IFNγ antibodies after stimulation, was enriched in PD-1^+^ T cells (Extended Data Fig. 3c). Analysis of cytokine production revealed no reduced ability of PD-1^int^ or PD-1^high^ aortic IFNγ^+^ CD4 or CD8 T cells to co-produce TNF and IL-2 compared to PD-1^−^ (Fig. 3c–f). Tox is a transcription factor expressed by both exhausted T cells and polyfunctional effector T cells^18^. We observed elevated levels of Tox in PD-1^high^ aortic T cells compared to PD-1^−^ counterparts (Extended Data Fig. 3f,g). Likewise, both aortic IFNγ-producing PD-1-expressing CD4 and CD8 T cells displayed a memory phenotype (Ly6C^−^PSGL^+/−^ and IL-7Rα^+^KLRG1^+/−^ for CD4 and CD8, respectively)^19,20^. In addition, we observed increased proportions of short-lived effector-like IL-7Rα^−^KLRG1^+/−^ T cells among IFNγ^+^ PD-1^high^ CD8 T cells (Extended Data Fig. 3h–k). Altogether, aortic PD-1^+^ T cells were not functionally exhausted, retaining the capacity to proliferate and produce several cytokines upon restimulation.

### Aortic PD-1^+^ T cells express CD69 and show signs of recent TCR signaling

**Fig. 4:**
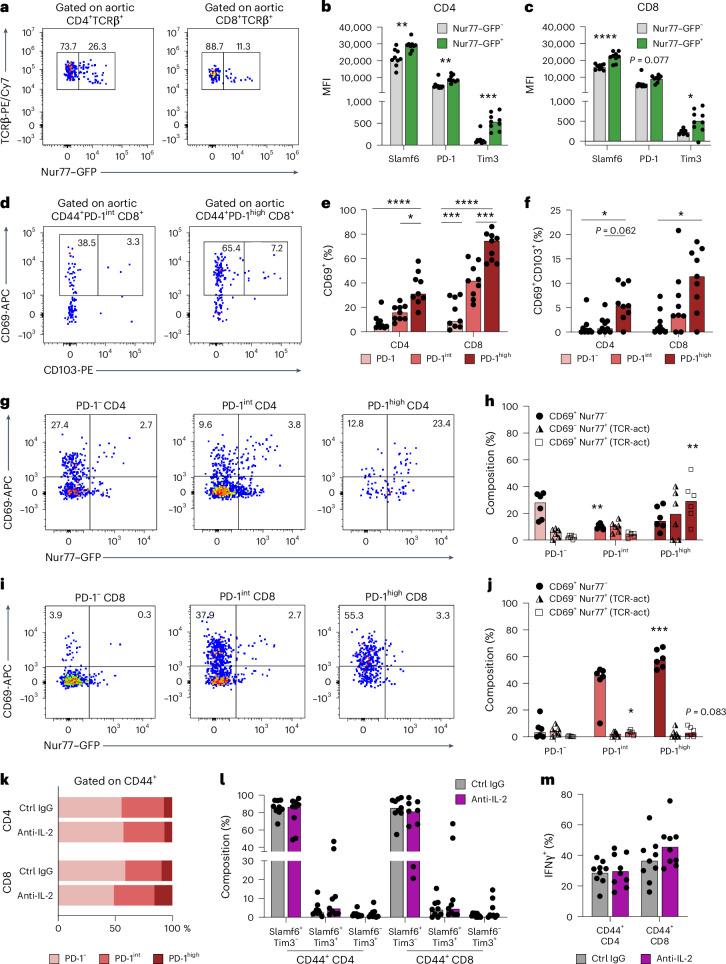
Aortic PD-1^+^ T cells express CD69 and show signs of recent TCR signaling. **a**–**c**, TCR signaling reporter mice (*Nur77*^*wt/GFP*^*Apoe*^−*/*−^) were generated and fed an HCD for 12 weeks, and, at euthanization, T cell phenotype was analyzed (*n* = 9). **a**, Flow cytometry plots of Nur77–GFP expression on aortic T cells. **b**, MFI of Slamf6 (**P = 0.004), PD-1 (**P = 0.0019) and Tim3 (***P = 0.0002) of Nur77^−^ and Nur77^+^ (recent TCR activation) aortic CD4. **c**, MFI of Slamf6 (****P = 9.2 × 10^−5^), PD-1 and Tim3 (*P = 0.01) of Nur77^−^ and Nur77^+^ aortic CD8 T cells. **d**, Flow cytometry plots of CD69 and CD103 expression on aortic CD8 T cells of *Ifng*^*YFP/YFP*^*Apoe*^−*/*−^ mice fed an HCD for 14 weeks (*n* = 9). **e**,**f**, Quantification of percent CD69^+^ T cells (CD4 ****P = 8.5 × 10^−5^, *P = 0.049; CD8 ****P = 5.6 × 10^−9^, ***P = 0.0004) and CD69^+^CD103^+^ T cells (CD4 *P = 0.012, CD8 *P = 0.011) within PD-1 subsets in the aorta. **g**,**h**, Flow cytometry analyzing CD69 and Nur77–GFP expression within PD-1 subsets of aortic CD4 T cells from *Ifng*^*YFP/YFP*^*Nur7*^*wt/GFP*^*Apoe*^−*/*−^ mice fed an HCD for 10 weeks (*n* = 6). Quantification of non-activated CD4 T_RM_-like cells (CD69^+^Nur77–GFP^−^) and recently activated CD4 T cells (CD69^−^Nur77–GFP^+^ and CD69^+^Nur77–GFP^+^). **i**–**j**, Flow cytometry analyzing CD69 and Nur77–GFP expression within PD-1 subsets of aortic CD8 T cells. **k**–**m**, In a separate cohort, *Ifng*^*YFP/YFP*^*Apoe*^−*/*−^ mice were fed an HCD for 10 weeks before receiving intraperitoneal injections of anti-IL-2 antibodies or control IgG (*n* = 9 per group) for 2 weeks. **k**,**l**, Flow cytometric analysis of aortic memory T cells to assess composition of PD-1 subsets and subsets of cells expressing Slamf6 and/or Tim3. **m**, Flow cytometric analysis of IFNγ production by aortic CD44^+^CD4 and CD44^+^CD8 T cells. **b**,**l**, Bars denote median, analyzed with two-sided Mann–Whitney *U*-test. **c**,**m**, Bars denote mean, analyzed with two-sided unpaired *t*-test. **e**,**f**,**h**,**j**, Bars denote median, analyzed with two-sided Kruskal–Wallis test. **h**,**j**, *P* values compared to PD-1^−^ subset (CD4 PD-1^int^ **P = 0.0088, CD4 PD-1^high^ *P = 0.0016, CD8 PD-1^int^ *P = 0.031, CD8 PD-1^high^ ***P = 0.0005). act, activated; Ctrl, control. Source Data

**Extended Data Fig. 4:**
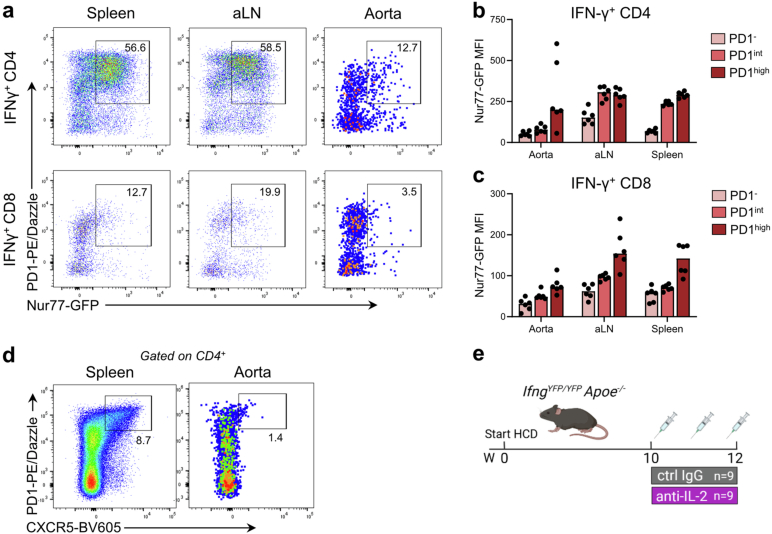
PD1 expression in relation to T-cell receptor signaling and IL-2 signaling. (**a**-**c**) Atherosclerotic dual-reporter mice (*Ifng*^*YFP/YFP*^*Nur77*^*wt/GFP*^*Apoe*^−*/*−^) were generated, fed a high-cholesterol diet for 10 weeks, and flow cytometry was performed to characterize Nur77 expression on IFN-γ-producing T cells in spleen, iliac aortic-draining lymph node (aLN), and aorta (n = 6). (**a**) Flow cytometry plots and (**b-c**) quantification of association between Nur77-GFP expression and PD1 expression within IFN-γ-YFP^+^ CD4 and CD8 T cells in the aorta, iliac aortic-draining lymph nodes (aLN), and spleen. (**d**) Representative flow cytometry gating for identifying follicular T helper cells (Tfh; CXC5^+^PD1^high^ CD4^+^) in *Ifng*^*YFP/YFP*^*Apoe*^−*/*−^ mice. (**e**) Experimental design for anti-IL-2 study. *Ifng*^*YFP/YFP*^*Apoe*^−*/*−^ mice were fed a high cholesterol diet (HCD) for 10 weeks before receiving *i.p*. injections (3 doses, 5 days apart) of anti-IL-2 antibodies or control IgG (n = 9/group) for 2 weeks. Source Data

PD-1 is transiently upregulated in response to recent TCR signaling and remains elevated on progenitor or late exhausted T cells^12^. To explore if expression of exhaustion-related markers was associated with recent TCR signaling, we analyzed T cells isolated from *Nur77*^*wt/GFP*^*Apoe*^−/−^ reporter mice (Fig. 4a). In these mice, green fluorescent protein (GFP) production in T cells is proportional to the amount of recent TCR signaling, reflecting priming, reactivation or tonic signaling of T cells^18^. Supporting recent TCR activation in aortic PD-1^+^ T cells, surface-level expression of PD-1, Slamf6 and Tim3 was higher on aortic Nur77–GFP^+^ CD4 and CD8 T cells compared to Nur77–GFP^−^ counterparts (Fig. 4b,c). Similarly, evaluating aortic T cells from HCD-fed *Ifng*^*YFP/YFP*^*Nur7*^*wt/GFP*^*Apoe*^−*/*−^ mice, we confirmed a positive association between Nur77 MFI and graded PD-1 expression on IFNγ-producing T cells in the aorta (Extended Data Fig. 4a–c).

PD-1 is also expressed in other T cell subsets, including CXCR5^+^ T follicular helper (T_FH_) cells and CD69^+^CD103^+/−^ tissue-resident memory (T_RM_) cells^21^. Analyzing aortas from HCD-fed *Ifng*^*YFP/YFP*^*Apoe*^−*/*−^ mice, we observed little expression of CXCR5 on aortic PD-1^+^CD4^+^ T cells, indicating that these cells were distinct from lymphoid tissue T_FH_ cells (Extended Data Fig. 4d). To characterize the T_RM_-like phenotype of aortic PD-1^+^ T cells, we performed flow cytometric analysis for CD69 and CD103 (Fig. 4d). We observed a stepwise increase in levels of CD69^+^ and, to a limited extent, double-positive CD69^+^CD103^+^ in PD-1^int^ and PD-1^high^ T cells, indicating overlap between early exhaustion and T_RM_-like phenotype (Fig. 4e,f).

CD69 is expressed on both recently activated T cells and T_RM_ cells. To differentiate these populations, we analyzed CD69 and Nur77–GFP on aortic T cells from HCD-fed *Ifng*^*YFP/YFP*^*Nur7*^*wt/GFP*^*Apoe*^−*/*−^ mice. Four distinct populations were identified: (1) non-activated, non-T_RM_-like cells; (2) non-activated T_RM_-like cells (Nur77–GFP^−^CD69^+^); (3) recently activated T cells (Nur77–GFP^+^CD69^+^); and (4) activated T cells not upregulating CD69 (Nur77–GFP^+^CD69^−^). We found that PD-1^high^ CD4 T cells displayed signs of recent TCR activation (Nur77–GFP^+^CD69^+^) compared to PD-1^−^ and PD-1^int^ subsets (Fig. 4g,h). For CD8 T cells, we observed upregulation of Nur77–GFP and CD69 in PD-1^int/high^ T cells, however at markedly lower levels of positivity compared to plaque CD4 T cells. Notably, the majority of PD-1^int^ and PD-1^high^ CD8 T cells were positive for CD69 without concomitant Nur77–GFP expression (Fig. 4i-j).

To explore if the exhaustion phenotype of aortic T cells in atherosclerotic mice was a consequence of excessive IL-2 signaling, we injected *Ifng*^*YFP/YFP*^*Apoe*^−*/*−^ mice with blocking monoclonal anti-IL-2 antibodies or isotype control IgG (Extended Data Fig. 4e). However, short-term IL-2 blockade did not affect PD-1 subset composition of CD4 or CD8 T cells, patterns of SLAMF6/Tim3 expression or IFNγ production in plaques (Fig. 4k–m). Collectively, these results indicate that PD-1 expression on aortic T cells, especially CD4 T cells, is associated with recent TCR signaling and that aorta-residing PD-1^+^ CD8 T cells are characterized by CD69 expression.

### Plaque-antigen expanded T cells express TOX

**Fig. 5:**
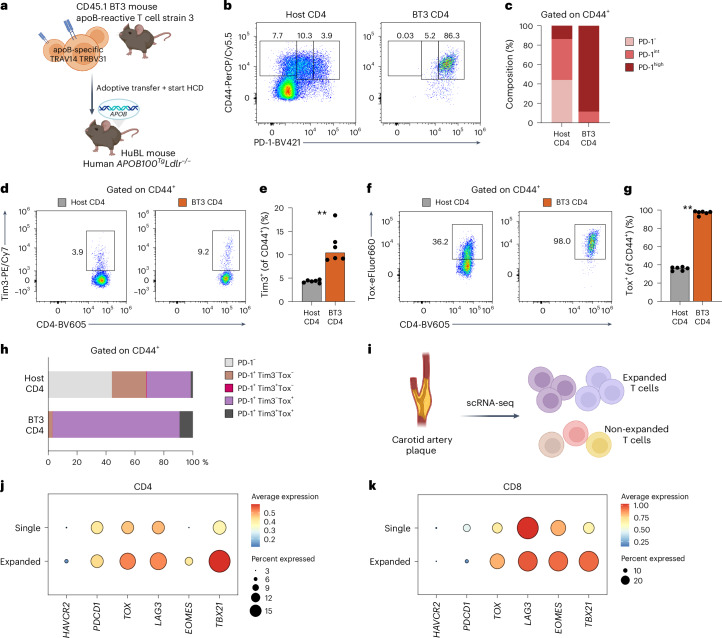
Plaque-antigen expanded T cells express TOX. **a**, Congenic CD4 T cells reactive against a peptide sequence of human apolipoprotein B-100 (apoB-100; TRBV31^+^ CD4 T cells) from CD45.1^+^ BT3 mice were transferred into atherosclerotic mice that produce human apoB-100 (*APOB100*^*Tg*^*Ldlr*^−*/*−^; HuBL mouse, ‘Host’). HuBL mice were fed an HCD for 3 weeks after adoptive transfer and then euthanized (*n* = 6 per group). **b**,**c**, Representative flow cytometry plots of PD-1 expression and quantification of PD-1 subsets within host CD4 T cells or transgenic BT3 CD4 T cells in spleens of recipient HuBL mice. Representative flow cytometry plots and quantification of Tim3^+^ (**P = 0.0022) (**d**,**e**) and Tox (**P = 0.0022) (**f**,**g**) expression within transgenic (BT3) and non-transgenic (Host) memory CD4 T cells in spleen of HuBL mice. **h**, T cell composition comparing non-transgenic (Host) and transgenic (BT3) CD44^+^CD4 T cells in the spleen. **i**, Atherosclerotic plaques from carotid endarterectomy patients were analyzed by scRNA-seq to evaluate gene expression differences between non-expanded (single clone) compared to expanded (≥2 clones) T cells (*n* = 3). **j**,**k**, Average expression of T cell exhaustion-related genes in plaque CD4 and CD8 T cells. **e**,**g**, Bars denote median, analyzed with two-sided Mann–Whitney *U*-test. Source Data

**Extended Data Fig. 5:**
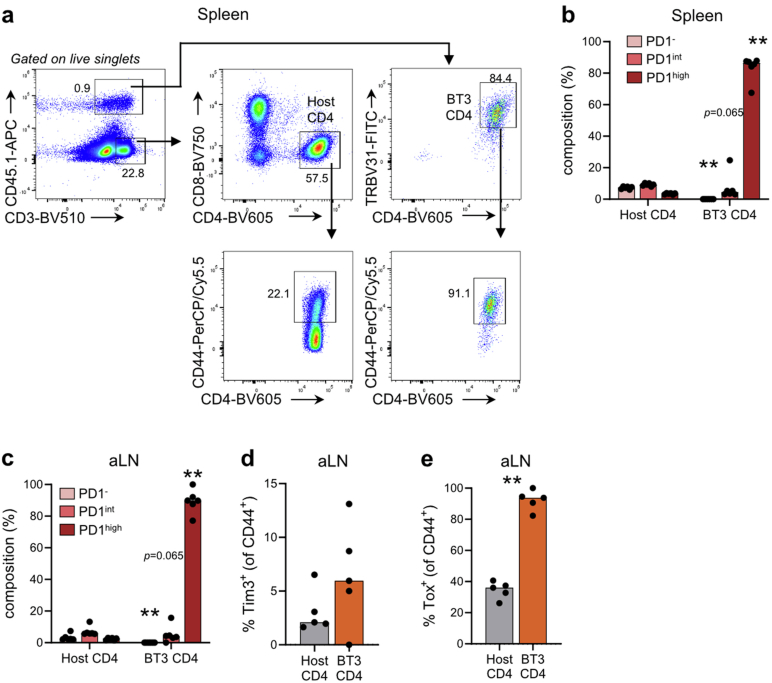
Exhaustion phenotype of ApoB-100-specific transgenic T cells. Transgenic and congenic (CD45.1) CD4 T cells (BT3) reactive against a peptide sequence of human apolipoprotein B-100 (apoB-100; TRBV31^+^ CD4 T cells) were transferred into atherosclerotic mice that produce human apoB-100 (*APOB100*^*Tg*^*Ldlr*^−*/*−^; HuBL mouse). HuBL mice were fed a high-cholesterol diet for three weeks after adoptive transfer and then terminated (n = 6/group). (**a**) Gating strategy identifying CD44^+^ host CD4 and CD45.1^+^ BT3 T cells. (**b-c**) Quantification of PD1 subset composition comparing host CD4 and BT3 T cells in spleen (**p = 0.0022) and aortic-draining iliac lymph node (aLN, **p = 0.0022). Frequencies of (**d**) CD44^+^Tim3^+^ CD4 T cells and (**e**) CD44^+^Tox^+^ CD4 T cells within host and BT3 CD4 T cells in aLN (**p = 0.0079). (**b-e**) Bars denote median, analyzed with two-sided Mann-Whitney *U* test. Source Data

Autoimmunity against plaque antigens has been observed in mouse models of atherosclerosis^15,22^ and in humans^23^. To investigate the exhaustion phenotype of T cells specific for plaque antigen, we used CD45.1 congenic apoB-reactive TCR transgenic mice (apoB-reactive T cell stain 3; BT3) where TRBV31^+^ CD4 T cells respond against a peptide sequence of human apolipoprotein B-100 (apoB-100)^24^. We transferred CD4 T cells from CD45.1^+^ BT3 mice into atherosclerotic mice that produce human apoB-100 (*APOB100*^*Tg*^*Ldlr*^−*/*−^; HuBL mouse). HuBL recipient mice were fed an HCD for 3 weeks after transfer until euthanization and analysis of apoB-reactive CD4 T cells (Fig. 5a and Extended Data Fig. 5a). Reflecting chronic activation of apoB-100-specific T cells in hypercholesterolemic mice, transgenic CD4 T cells (CD45.1^+^TRBV31^+^ from BT3 donors) exhibited high PD-1 expression compared to non-transgenic memory CD4 T cells (CD44^+^CD45.1^−^) in both spleen (*P* = 0.0022; Fig. 5b,c and Extended Data Fig. 5b) and aorta-draining iliac lymph nodes (*P* = 0.0079; Extended Data Fig. 5c). Splenic apoB-specific BT3 T cells displayed increased levels of Tim3^+^ expression (*P* = 0.0022) as well as markedly elevated levels of the transcription factor Tox (*P* = 0.0022) (Fig. 5d–h), with similar trends observed in lymph nodes (Extended Data Fig. 5d,e).

We next tested if expanded T cell clones in human plaques, likely containing both putative plaque antigen-specific T cells as well as T cells reactive against pathogens, displayed a phenotype reminiscent of apoB-100 autoreactive transgenic BT3 T cells. To this end, we analyzed scRNA-seq data of human carotid plaques^15^ and compared gene expression in non-expanded (single clone) to expanded (≥2 clones) CD4 and CD8 T cells (Fig. 5i). Although we did not observe robust *PDCD1* expression, both expanded CD4 and CD8 T cells displayed increased expression of several genes associated with effector function and exhaustion, including the genes *TOX*, *EOMES* and *TBX21* as well as *LAG3* in CD8 T cells (Fig. 5j,k).

### PD-1 blockade shifts aortic T cells toward a PD-1^high^ phenotype and increases IFNγ production

**Fig. 6:**
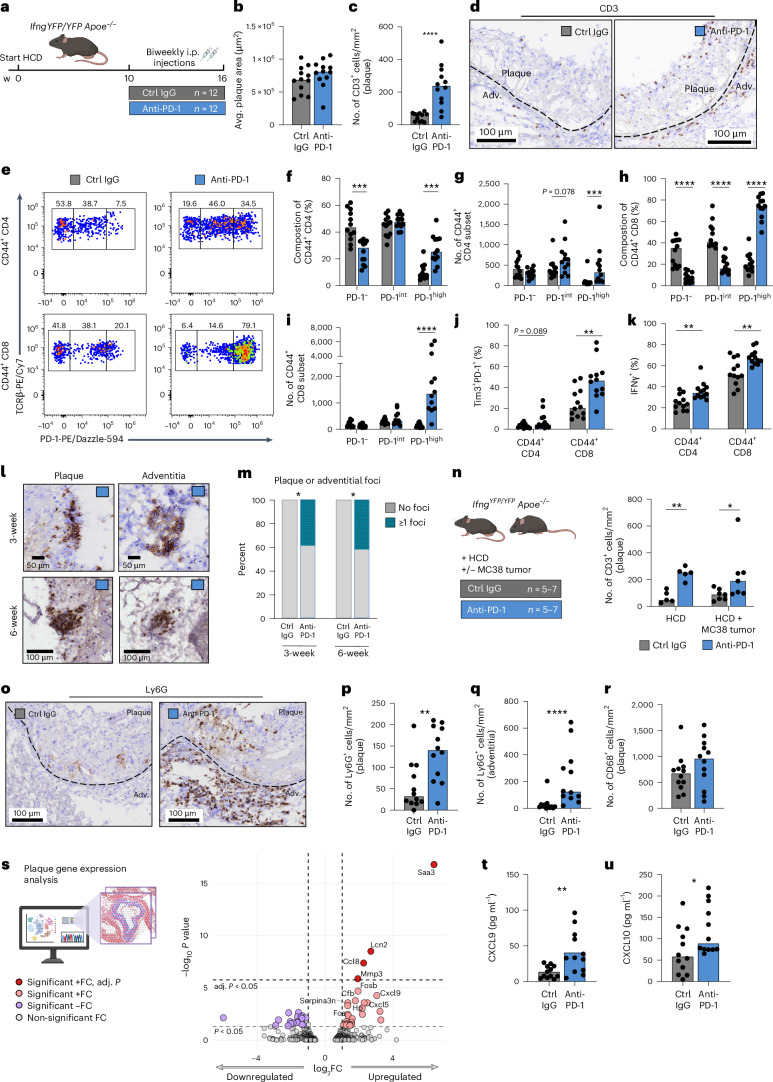
Anti-PD-1 treatment increases T cell plaque infiltration and IFNγ production by aortic T cells. **a**–**k**,**o**–**u**, *Ifng*^*YFP/YFP*^*Apoe*^−*/*−^ mice were fed an HCD for 10 weeks before administering biweekly intraperitoneal injections of a murine blocking anti-PD-1 antibody or isotype IgG control (*n* = 12 per group). Mice received a total of 12 injections over 6 weeks and were euthanized after a total of 16 weeks of diet. **b**, Quantification of average plaque area of aortic subvalvular cross-sections. **c**,**d**, Quantification and representative immunohistochemical staining of T cells (CD3^+^) in aortic root plaques (****P = 5 × 10^−5^). **e**–**i**, Representative flow cytometry plots and quantification of frequency and numbers of PD-1 subsets of aortic memory CD4 (***P = 0.0001) and CD8 (**h** ****P = 7.4 × 10^−7^, i****P = 5.2 × 10^−6^) T cells. **j**,**k**, Flow cytometric analysis of frequency of Tim3^+^PD-1^+^ memory CD4 and CD8 aortic T cells (**P = 0.0024) and frequency of IFNγ production of memory CD4 and CD8 aortic T cells (**P = 0.0023, 0.0022). **l**,**m**, Representative immunohistochemical images of and quantification of presence of at least one T cell foci in the plaque and/or surrounding adventitia (*P = 0.024). For study details of 3-week anti-PD-1 cohort, see Extended Data Fig. 6 (ctrl IgG *n* = 12, anti-PD-1 *n* = 13). **n**, Impact of presence of tumor (MC38 tumor) during anti-PD-1 therapy (*n* = 7 per group) on T cell (CD3^+^) infiltration into subvalvular aortic plaques (**P = 0.0079, *P = 0.026) compared to ctrl IgG (*n* = 5 per group), determined by immunohistochemistry. **o**–**q**, Quantification and representative immunohistochemical staining of neutrophils (Ly6G^+^) in aortic root plaques (**P = 0.0070) and adventitia (****P = 9.5 × 10^−5^) in *Ifng*^*YFP/YFP*^*Apoe*^−*/*−^ mice treated with PD-1 blockade or isotype IgG ctrl for 6 weeks (*n* = 12 per group). **r**, Quantification of immunohistochemical staining of macrophages (CD68^+^) in aortic root plaques. **s**, Gene expression analysis of aortic subvalvular cross-sections of ctrl IgG-treated or anti-PD-1-treated mice. Genes are reported as upregulated or downregulated in anti-PD-1-treated mouse compared to isotype IgG control. Adjusted *P* value is adjusted for FDR. **t**,**u**, Concentration of plasma cytokines CXCL9 (**P = 0.0063) and CXCL10 (*P = 0.037). **b**,**c**,**i**,**n**,**p**–**r**,**u**, Bars denote median, analyzed with two-sided Mann–Whitney *U*-test. **t**, Bars denote mean, analyzed with two-sided unpaired *t*-test. **f**–**h**,**j**,**k**, Bars denote median, analyzed with two-sided Mann–Whitney *U*-test or two-sided unpaired *t*-test. **m**, Analyzed with one-sided Fisher’s exact test. adj., adjusted; Adv., adventitia; Avg., average; ctrl, control; FC, fold change; i.p., intraperitoneal; w, week. Source Data

**Extended Data Fig. 6:**
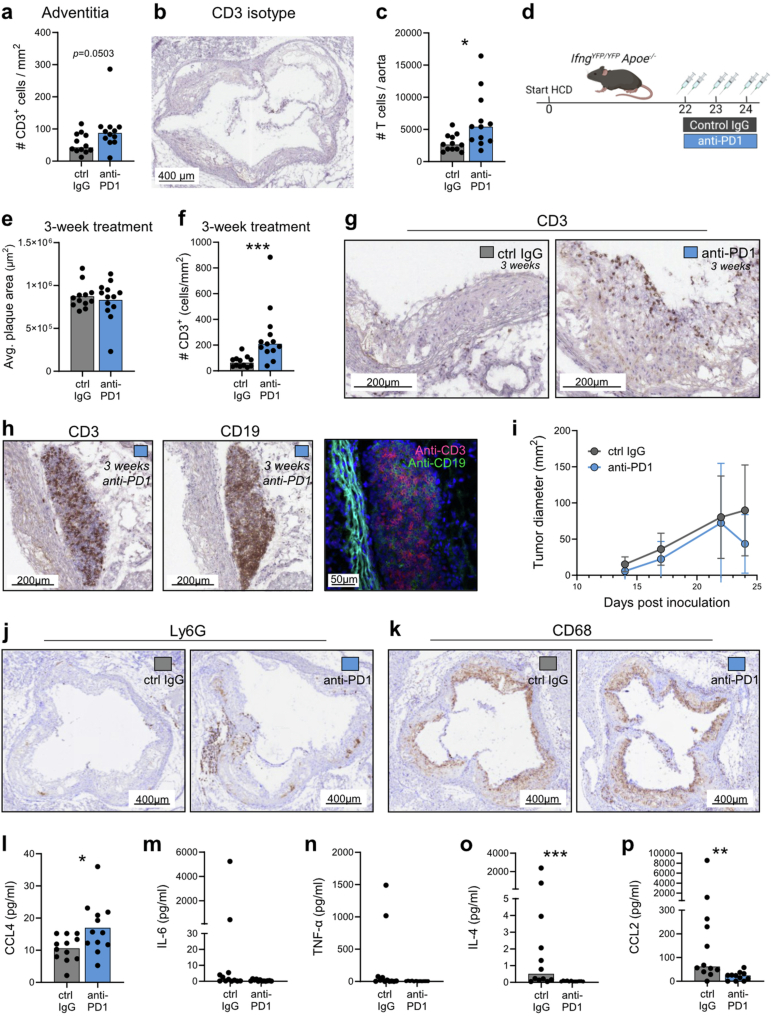
Impact of anti-PD1 blockade on plaque inflammation and plasma chemokines/cytokines. *Ifng*^*YFP/YFP*^*Apoe*^−*/*−^ mice were fed a high-cholesterol diet for 10 weeks before administration of bi-weekly *i.p*. injections of a murine blocking anti-PD1 antibody or isotype IgG control (n = 12/group) for 6 weeks. (**a**) Quantification of CD3^+^ cells in adventitia of aortic-root sections. Adventitia designated as 100 μm from smooth muscle layer of aortic valve. (**b**) Isotype IgG staining for CD3 immunohistochemical analysis of aortic root cross-sections. (**c**) Quantification of T cells (TCRβ^+^CD45^+^) per whole-aorta digest analyzed by flow cytometry (*p = 0.010). (**d-h**) In the short-term anti-PD1 cohort, *Ifng*^*YFP/YFP*^*Apoe*^−*/*−^ mice were fed a high-cholesterol diet for 21 weeks before administration of bi-weekly *i.p*. injections of anti-PD1 antibody or isotype IgG control (n = 11-12/group). Mice received a total of six injections and were terminated after a total of 24 weeks of diet. (**e**) Average plaque area determined by Oil Red O staining of aortic subvalvular cross-sections. (**f-g**) Quantification and representative image of immunohistochemical analysis of CD3^+^ cells in aortic root plaques (***p = 0.0002). (**h**) Representative immunohistochemical staining images and confocal images of CD3 and CD19 staining of advanced adventitial foci. (**i**) Measurements of MC38 tumor diameter in HCD-fed *Ifng*^*YFP/YFP*^*Apoe*^−*/*−^ mice with (n = 5) and without (n = 7) PD1 blockade. Data presented as mean values +/- SD. (**j-k**) Representative images of immunohistochemical staining of neutrophils (Ly6G) and macrophages (CD68) in *Ifng*^*YFP/YFP*^*Apoe*^−*/*−^ mice treated with PD1 blockade or isotype IgG control for 6 weeks (n = 12/group). (**l-p**) Concentration of plasma cytokines CCL4 (*p = 0.023), IL-6, TNF-α, IL-4 (***p = 0.0009), and CCL2 (**p = 0.0013) in *Ifng*^*YFP/YFP*^*Apoe*^−*/*−^ mice treated with anti-PD1 or isotype IgG control for 6 weeks. (**a**, **c**, **f**, **m**-**p**). Bars denote median, analyzed with two-sided Mann-Whitney *U* test. (**e, l**) Bars denote mean, analyzed with two-sided unpaired *t* test. Source Data

Recent studies have demonstrated an increased risk of atherosclerotic cardiovascular events in patients treated with anti-PD-1 ICIs^8,10,25^, but understanding of how aortic T cell phenotype is affected by this treatment is lacking. To address this question, we administered biweekly injections of anti-PD-1 antibody or isotype IgG control, for a total of 6 weeks, to *Ifng*^*YFP/YFP*^*Apoe*^−*/*−^ mice fed an HCD for 10 weeks prior to PD-1 blockade (Fig. 6a). To this end, we used a murinized version of the RMP1.14 anti-PD-1 antibody to minimize emergence of antibodies against the injected anti-PD-1 antibody. In accordance with previous reports^4^, we observed no difference in plaque size in aortic valve cross-sections in mice treated with anti-PD-1 compared to control mice (Fig. 6b). Immunohistochemical staining with anti-CD3 demonstrated a dramatic influx of T cells to plaques of anti-PD-1-treated mice (*P* = 5 × 10^−5^; Fig. 6c,d) and a trend toward increased numbers of adventitial T cells (Extended Data Fig. 6a,b). Accordingly, flow cytometric analysis demonstrated increased numbers of aortic T cells after PD-1 blockade (Extended Data Fig. 6c).

Next, we assessed how PD-1 blockade affected T cell composition in the atherosclerotic aorta. We observed a dramatic shift of memory T cells toward a PD-1^high^ phenotype in mice treated with anti-PD-1 (Fig. 6e–i). This effect was the most pronounced for CD8 T cells with approximately 75% PD-1^high^ T cells in anti-PD-1-treated mice compared to approximately 20% PD-1^high^ T cells in isotype control-treated mice (Fig. 6h). Progenitor exhausted PD-1^int^ T cells, capable of differentiating into PD-1^high^ T cells in response to ICI therapy^11^, remained at equal numbers in the aorta (Figs. 6g,i). Treatment with anti-PD-1 has been associated with increased frequency of late exhausted PD-1^+^Tim3^+^ T cells in murine tumors^18^. Similarly, we observed significantly increased levels of PD-1^+^Tim3^+^ CD8 T cells (*P* = 0.0024) and a trend for increased levels of PD-1^+^Tim3^+^ CD4 T cells (*P* = 0.089) in atherosclerotic aortas after anti-PD-1 treatment (Fig. 6j), suggesting increased generation of PD-1^+^Tim3^+^ late exhausted CD8 T cells from progenitor exhausted CD8 T cells. These changes were accompanied by an increase in YFP^+^ IFNγ-producing T cells after PD-1 blockade (Fig. 6k). By immunohistochemical analysis, we observed formation of CD3^+^ T cell foci in aortic sinus plaque and adventitia (*P* = 0.024) compared to control IgG-treated mice where none was observed (Fig. 6l,m). Development of lymphocyte foci was replicated in a cohort of *Ifng*^*YFP/YFP*^*Apoe*^−*/*−^ mice treated with anti-PD-1 for 3 weeks (Fig. 6l,m and Extended Data Fig. 6d–h). In one advanced adventitial T cell foci, we observed accumulation of adjacent CD19^+^ B cells (Extended Data Fig. 6h), suggesting early tertiary lymphoid structure formation. In a separate cohort (*n* = 5–7 mice per group), we validated that concurrent MC38 tumor burden does not significantly alter anti-PD-1-driven recruitment of T cells to plaques (Fig. 6n and Extended Data Fig. 6i).

To evaluate how anti-PD-1 therapy impacts myeloid cell accumulation in plaques, we performed immunohistochemical assessment of Ly6G (neutrophils) and CD68 (macrophages). We observed elevated levels of Ly6G^+^ neutrophils in both plaque and adventitia in anti-PD-1-treated mice (Fig. 6o–q and Extended Data Fig. 6j) but no significant change in levels of CD68^+^ plaque macrophages (Fig. 6r and Extended Data Fig. 6k). To gain additional insight into how PD-1 blockade impacts plaque phenotype, we performed spatial transcriptomic analysis comparing control IgG-treated and anti-PD-1-treated mice (Fig. 6s). Differential gene expression analysis revealed several significantly upregulated genes in anti-PD-1-treated plaques: *Saa3*, *Lcn2*, *Ccl8* and *Mmp3*. In addition, we also observed increased transcription of genes associated with T cell and neutrophil chemotaxis (*Cxcl5* and *Cxcl9*), although not reaching the significance threshold when adjusting for multiple testing (Supplementary Table 2). Analyzing plasma, we found elevated levels of the IFNγ-inducible cytokines CXCL9 and CXCL10 (Fig. 6t,u) as well as the CCR5 ligand CCL4 (Extended Data Fig. 6l) in anti-PD-1-treated mice. Plasma levels of IL-6 and TNF were not affected by anti-PD-1 treatment, and we observed decreased levels of both IL-4 and CCL2 (Extended Data Fig. 6m–p). Altogether, anti-PD-1 treatment promoted a dramatic accumulation of PD-1^high^ late exhausted T cells in plaques and increased levels of IFNγ production, creating a pro-inflammatory milieu as demonstrated by chemokine production and increased neutrophil influx.

### Pre-depletion of PD-1^high^ T cells does not impair anti-PD-1-driven accumulation of plaque T cells

**Fig. 7:**
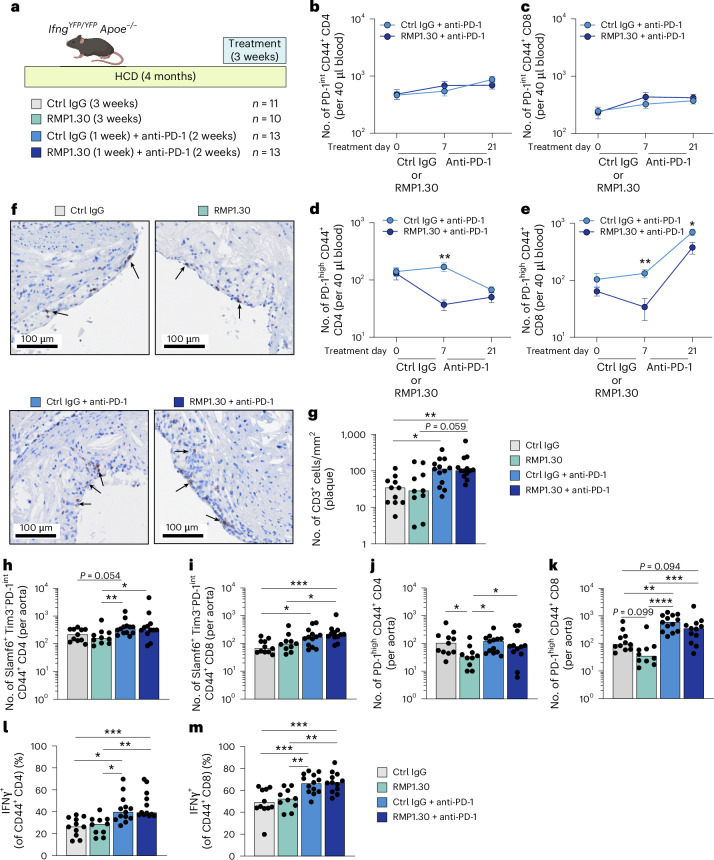
Pre-depletion of PD-1^high^ T cells does not impair anti-PD-1-driven accumulation of plaque T cells. *Ifng*^*YFP/YFP*^*Apoe*^−*/*−^ mice were fed with an HCD for 4 months and treated for the final 3 weeks with isotype control only (*n* = 11), depleting anti-PD-1 (RMP1.30, *n* = 10) only, ctrl IgG for 1 week prior to injection of blocking anti-PD-1 antibodies (ctrl IgG + anti-PD-1, *n* = 13) or depleting anti-PD-1 (RMP1.30) for 1 week prior to injection of blocking anti-PD-1 antibodies (RMP1.30 + anti-PD-1, *n* = 13). **a**, Experimental overview. **b**,**c**, Numbers of circulating CD44^+^PD-1^int^ CD4 and CD8 T cells throughout treatment course (days 0, 7 and 21). **d**,**e**, Numbers of circulating CD44^+^PD-1^high^ CD4 (**P = 0.0010) and CD8 (**P = 0.0014, *P = 0.017) T cells throughout the treatment course. **f**,**g**, Quantification and representative immunohistochemical staining of T cells (CD3^+^) in aortic root plaques (**P = 0.0077, *P = 0.027). **h**,**i**, Flow cytometric analysis of counts of progenitor exhausted (CD44^+^Slamf6^+^Tim3^−^PD-1^int^) aortic CD4 (*P = 0.025, **P = 0.0072) and CD8 (***P = 0.0004, *P = 0.014, 0.012) T cells. **j**,**k**, Flow cytometric analysis of counts CD44^+^PD-1^high^ aortic CD4 (*P = 0.039, 0.011, 0.014) and CD8 (***P = 0.009, 0.0021, ****P = 2.4 × 10^−6^) T cells. **l**,**m**, Flow cytometric analysis of frequency of IFNγ production by aortic memory CD4 (***P = 0.0006, **P = 0.0013, *P = 0.014, 0.026) and CD8 (***P = 0.0006, 0.0009, **P = 0.0061, 0.0097) T cells. **b**–**e**, Data presented as mean values ± s.e.m., analyzed with mixed-effects multiple comparisons analysis. **g**–**l**, Bars denote median, analyzed with Kruskal–Wallis test. **m**, Bars denote mean, analyzed with one-way ANOVA. *P* values are reported top-to-bottom and left-to-right. ctrl, control. Source Data

Previous studies in cancer and autoimmunity highlighted PD-1^int^ progenitor exhausted T cells, rather than late exhausted PD-1^high^ T cells, as cellular targets of immune checkpoint inhibition^18,26^. Inhibition of PD-1 signaling on PD-1^int^ T cells has been shown to promote proliferation and differentiation into PD-1^high^ T cells in tumors^11^. However, it is not evident which PD-1 subset (PD-1^int^ or PD-1^high^) is responsible for promoting anti-PD-1-driven T cell accumulation in atherosclerotic plaques. To address this question, we injected HCD-fed *Ifng*^*YFP/YFP*^*Apoe*^−*/*−^ mice with three doses of non-blocking PD-1^high^ depleting antibodies (clone RMP1.30, days 0, 3 and 6)^27^ to remove PD-1^high^ T cells before administering anti-PD-1 blocking antibody for 2 weeks (clone RMP1.14) until euthanization. This design allowed us to test whether PD-1^high^ T cells are necessary for anti-PD-1-driven T cell accumulation in plaques. To control for the effects of PD-1^high^ depletion alone, we also included mice treated with RMP1.30 for all 3 weeks (Fig. 7a).

In mice receiving RMP1.30 before anti-PD-1, we detected efficient PD-1^high^ depletion on treatment day 7 and a subsequent boost in CD8 PD-1^high^ T cells after switching to anti-PD-1 therapy, whereas levels of circulating PD-1^int^ T cells were unaffected (Fig. 7b–e). Next, we tested if pre-depletion of PD-1^high^ T cells by RMP1.30 would mitigate plaque T cell accumulation in response to PD-1 blockade. Notably, although both groups of mice receiving anti-PD-1 for the final 2 weeks displayed elevated levels of plaque T cells, pretreatment with RMP1.30 did not disrupt PD-1 blockade-driven T cell accumulation (Fig. 7f,g).

**Extended Data Fig. 7:**
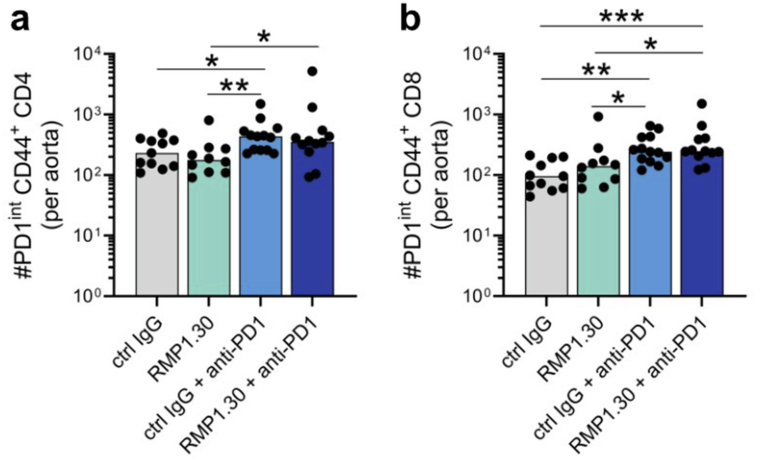
Pre-depletion of PD1^high^ T cells by anti-PD1 (clone RMP1.30) does not reduce numbers of aortic PD1^int^ T cells. *Ifng*^*YFP/YFP*^*Apoe*^−*/*−^ mice fed HCD-fed for 4 months were treated in the final 3 weeks with either: isotype control only (n = 11), depleting anti-PD1 (RMP1.30, n = 10) only, ctrl IgG for one week prior to injection of blocking anti-PD1 antibodies (ctrl IgG + anti-PD1, n = 13), depleting anti-PD1 (RMP1.30) for one week prior to injection of blocking anti-PD1 antibodies (RMP1.30 + anti-PD1; n = 13). (**a-b**) Quantification of aortic CD44^+^PD1^int^ CD4 (top-down, *p = 0.031, **p = 0.0044, *p = 0.035) and CD44^+^PD1^int^ CD8 (***p = 0.0005, *p = 0.019, **p = 0.0011, *p = 0.033) T cells. Bars denote median, analyzed with two-sided Kruskal-Wallis test. Source Data

Analyzing aortic digests by flow cytometry revealed no impact of RMP1.30 treatment in reducing the numbers of progenitor exhausted T cells defined as PD-1^int^Slamf6^+^Tim3^−^ T cells or CD44^+^PD-1^int^ (Fig. 7h,i and Extended Data Fig. 7a,b). Similar to what was observed in blood, we observed reductions in the numbers of aortic PD-1^high^ CD4 and CD8 T cells in mice receiving continuous RMP1.30 treatment (Fig. 7j,k). Of note, mice receiving transient RMP1.30 did not exhibit significantly lower numbers of PD-1^high^ T cells compared to PD-1 blockade without pre-depletion (Fig. 7j,k), likely due to expansion of PD-1^high^ T cells in response to PD-1 blockade. Anti-PD-1 treatment increased levels of IFNγ production of aortic T cells, but pre-depletion of PD-1^high^ T cells by RMP1.30 treatment did not ameliorate cytokine production (Fig. 7l,m). Altogether, these results demonstrate that PD-1^high^ T cells are not required for anti-PD-1-driven T cell accumulation in plaques and are consistent with a key role for PD-1^int^ T cells in orchestrating T cell accumulation in ICI-driven atherosclerosis.

### Human PD-1^+^ T cells produce IFNγ and are associated with presence of coronary atherosclerosis

**Fig. 8:**
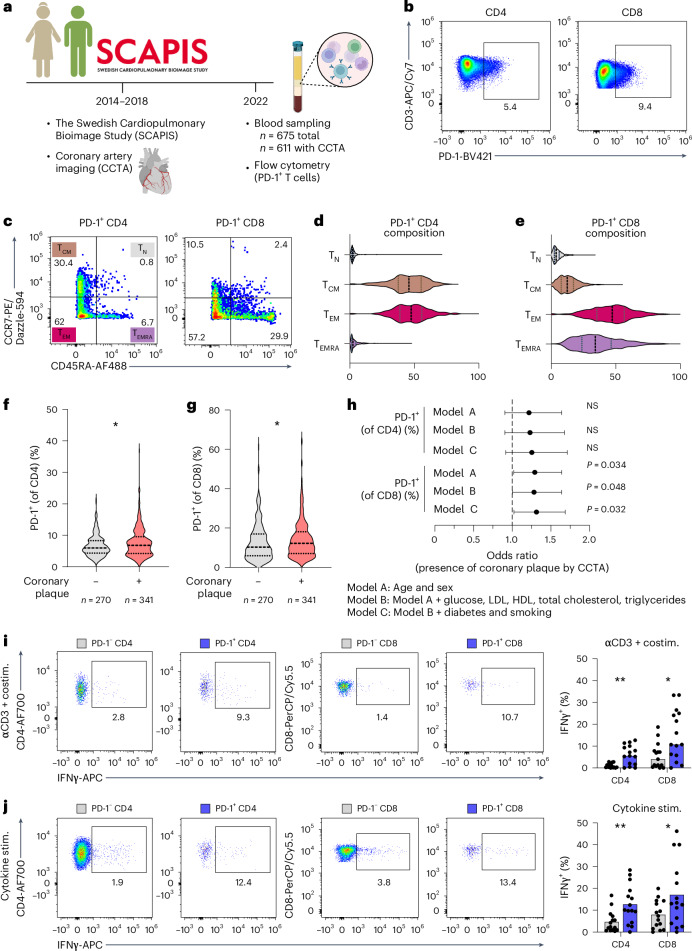
Human PD-1^+^ T cells produce IFNγ and are associated with presence of subclinical coronary atherosclerosis. **a**–**h**, PBMCs from 65–72-year-old individuals (*n* = 675) recruited from the general population who had previously been enrolled in the SCAPIS study were analyzed by flow cytometry. **a**, Study design. **b**–**e**, Representative flow cytometry plots and memory phenotype composition of PD-1^+^ circulating CD4 and CD8 T cells (T_N_, naive: CD45RA^+^CCR7^+^; T_CM_, central memory: CD45RA^−^CCR7^+^; T_EM_, effector memory: CD45RA^−^CCR7^−^; T_EMRA_, effector memory re-expressing CD45RA: CD45RA^+^CCR7^−^). **f**,**g**, Comparison of levels of expression of PD-1 on circulating CD4 (*P = 0.041) and CD8 (*P = 0.027) T cells between individuals with or without coronary plaque. **h**, Logistical regression analysis of PD-1 expression on circulating CD4 and CD8 T cells versus presence of subclinical coronary plaque (SIS ≥ 1; *n* = 611), adjusting for age and sex (Model A); Model A + glucose, LDL, HDL, total cholesterol and triglycerides (Model B); or Model B + diabetes and smoking (Model C). Error bars denote 95% confidence interval. **i**,**j**, PBMCs cultured for 24 hours in wells containing either anti-CD3 and co-stimulatory antibodies (anti-CD28/anti-CD49d) (**P = 0038, *P = 0.021) or stimulating cytokines (IL-12, IL-15 and IL-18) (**P = 0044, *P = 0.04) in the presence of brefeldin A, whereafter IFNγ production was assessed by intracellular flow cytometry. **f**,**g**, Violin plots, analyzed with two-sided Mann–Whitney *U*-test. **i**, Bars denote median, analyzed with two-sided Mann–Whitney *U*-test. **j**, Bars denote mean, analyzed with two-sided unpaired *t*-test. NS, non-significant; α, anti. Sgource Data

**Extended Data Fig. 8:**
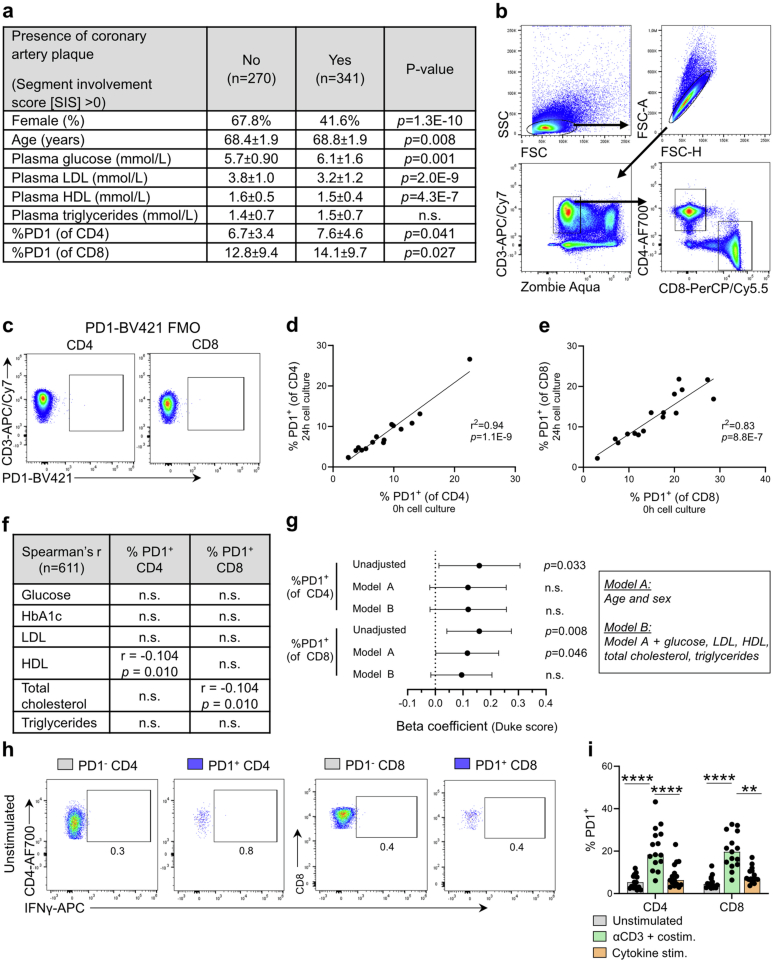
Circulating PD1^+^ T cells and subclinical atherosclerosis. (**a-g**) Blood from 65–72-year-old individuals (n = 675) recruited from the general population that had previously been enrolled in the Swedish Cardiopulmonary Bioimage Study (SCAPIS) was sampled. (**a**) Study population characteristics, metabolic profile, and levels of PD1^+^ T cells by flow cytometry. (**b-e**) Peripheral blood mononuclear cells were isolated and cultured in complete media with anti-CD28/anti-CD49d antibodies for 24 h and flow cytometry was performed. (**b**) Gating strategy to identify live CD4 and CD8 T cells. (**c**) Fluorescent minus-one (FMO) staining control for anti-PD1-BV421. (**d-e**) Correlation of %PD1^+^ T cells before and after 24-hour cell culture. (**f**) Correlation analysis of PD1 subsets and metabolic parameters (Spearman’s rank correlation co-efficient). (**g**) Beta co-efficients derived from linear regression analysis of association between PD1 subsets and Duke score (n = 611), unadjusted or adjusted for age and sex (Model A) or Model A + glucose, HDL, LDL, total cholesterol and triglycerides. Bars denote 95% CI and data was log transformed. (**h-i**) PBMCs cultured for 24 h in wells containing either anti-CD3 and co-stimulatory antibodies (anti-CD28/anti-CD49d) or stimulating cytokines (IL-12, IL-15, and IL-18) to study IFN-γ-production of human PBMCs were assessed by intracellular flow cytometry. (**h**) Negative controls (medium + Brefeldin A only). (**i**) Frequency of PD1 expression of CD4 (top-down, ****p = 5.2E-7, 1.7E-5) and CD8 T cells (****p = 1.2E-6, **p = 0.0067) in response to the different stimulation conditions. (**d-f**) Analyzed with two-sided Spearman’s rank correlation coefficients. (**i**) Bars denote median, CD4 T cells analyzed with two-sided one-way ANOVA and CD8 T cells analyzed with two-sided Mann-Whitney *U* test. Source Data

Our results in atherosclerotic mice indicated that PD-1^+^-expressing T cells may promote plaque inflammation. To test whether levels of circulating PD-1^+^ T cells were associated with atherosclerosis in humans, we analyzed blood samples from 65–72-year-old individuals (*n* = 675) recruited from the general population who had previously been enrolled in the Swedish Cardiopulmonary Bioimage Study (SCAPIS). Most of these individuals (*n* = 611) had undergone cardiac computed tomography angiography (CCTA) 4–8 years prior to a follow-up visit when blood was taken for peripheral blood mononuclear cell (PBMC) isolation, and clinical chemistry readouts (high-density lipoprotein (HDL), LDL, triglycerides and glucose) were assessed (Fig. 8a and Extended Data Fig. 8a). Cryopreserved PBMCs were thawed and cultured for 24 hours before flow cytometric analysis and assessment of PD-1 levels (Fig. 8b and Extended Data Fig. 8b). We validated specificity of staining by fluorescence minus one (FMO) controls for the anti-PD-1 antibody and that our cell culture conditions did not affect levels of PD-1 expression (Extended Data Fig. 8c–e). In circulating CD4 T cells, PD-1 expression was mainly restricted to T central memory (T_CM_) and T effector memory (T_EM_) subsets, whereas circulating PD-1^+^ CD8 T cells mainly comprised T_EM_ and T effector memory cells re-expressing CD45RA (T_EMRA_) subsets (Fig. 8c–e).

First, we evaluated associations between PD-1 subsets and metabolic readouts. Except for inverse correlations between total cholesterol and PD-1^+^ CD8 T cells (Spearmanʼs *ρ* = −0.104, *P* = 0.010) and PD-1^+^ CD4 T cells with HDL cholesterol (Spearmanʼs *ρ* = −0.100, *P* = 0.014), other metabolic parameters (LDL, triglycerides, glucose or hemoglobin A1c) were not associated with PD-1^+^ subsets (Extended Data Fig. 8f). As a measure of coronary atherosclerosis, we used the segment involvement score (SIS), a quantification of the number of coronary vessel segments containing atherosclerotic plaques regardless of plaque severity or stenosis, obtained from CCTA imaging. We stratified individuals by absence (SIS < 1, *n* = 270) or presence (SIS ≥ 1, *n* = 341) of coronary atherosclerosis. Levels of both PD-1^+^ CD4 (*P* = 0.041) and PD-1^+^ CD8 (*P* = 0.027) were significantly elevated in individuals with plaque compared to individuals without (Fig. 8f,g). The association between PD-1^+^ CD8 T cells and presence of plaque remained statistically significant after adjustment for age and sex (Model A, *P* = 0.034), adjustment for age, sex, glucose, LDL, HDL, total cholesterol and triglycerides (Model B, *P* = 0.048) and adjustment for diabetes and current smoking in addition to covariates listed in Model B (Model C, *P* = 0.032). The association between PD-1^+^CD4 T cells and presence of plaque did not remain statistically significant after adjustments (Fig. 8h). Additionally, we analyzed the association between the levels of PD-1^+^ T cells and Duke prognostic coronary artery disease score (Duke score), a composite score assessing plaque location and degree of stenosis^28^, by linear regression analysis. Results demonstrate an association between PD-1^+^ subsets and Duke score, especially for PD-1^+^ CD8 T cells for which the association with Duke score remained significant when adjusting for age and sex but not when adjusting for additional risk factors (Extended Data Fig. 8g). Neither PD-1^+^ CD8 T cells (*P* = 0.089, *ρ* = 0.066) nor PD-1^+^ CD4 T cells (*P* = 0.235, *ρ* = 0.046) were significantly associated with coronary calcium score.

Finally, we tested if human PD-1^+^ T cells had increased capacity for producing IFNγ, as we had observed in atherosclerotic mice. To this end, we cultured PBMCs from a subset of SCAPIS participants (*n* = 15), with (1) media alone, (2) agonistic anti-CD3 and co-stimulation or (3) T-cell-activating cytokines (IL-12/IL-15/IL-18) for 24 hours in the presence of brefeldin A during the last 5 hours and quantified production of IFNγ. We observed no difference in IFNγ production in unstimulated T cells (Extended Data Fig. 8h). Agonistic anti-CD3 and co-stimulation treatment promoted increased production of IFNγ in PD-1^+^ T cells (Fig. 8i) but, as expected, also led to de novo surface expression of PD-1 (ref. ^12^) (Extended Data Fig. 8i) on activated CD4 and CD8 T cells. However, innate stimulation by cytokines (bystander activation), which did not increase PD-1 expression, likewise demonstrated elevated rates of IFNγ production in PD-1-expressing T cells compared to PD-1^−^ T cells (Fig. 8j). These studies demonstrate that PD-1^+^ T cells can be activated by both classical TCR-mediated and cytokine-mediated activation, resulting in higher levels of IFNγ positivity compared to PD-1^−^ T cells.

## Discussion

ICI therapies that limit tumor growth in patients with cancer have been associated with increased risk of cardiovascular disease^8,9^. We demonstrate that PD-1 is expressed on aortic IFNγ-producing T cells and that the majority of the PD-1^+^ T cells in the atherosclerotic aorta display a polyfunctional progenitor exhausted state. Blocking PD-1 signaling increased T cell cytokine production, formation of lymphocyte foci in the plaque and accumulation of late exhausted PD-1^+^Tim3^+^ T cells. Suggesting a key role for progenitor exhausted PD-1^int^ T cells, the other main PD-1-expressing subset, PD-1^high^ T cells, was not required for the induction of T cell influx to plaques after PD-1 blockade. Human circulating PD-1^+^ T cells were enriched for cells capable of producing IFNγ upon restimulation and were associated with presence of subclinical atherosclerosis.

Repeated antigen encounter skews T cells toward a gradually more exhausted state characterized by muted response to antigen restimulation, limited capacity to produce cytokines and upregulation of immune checkpoint receptors^12^. Although T cell exhaustion has a detrimental role in limiting T cell responses against tumor cells, immune checkpoint receptors protect the host against autoimmunity^29,30^. The importance of PD-1 signaling in preserving tissue homeostasis is evident by the systemic inflammation and autoimmunity caused by inherited PD-1 deficiency^31^ or immune-related adverse events (irAEs) caused by anti-PD-1 ICI^32^. Our observation of a Slamf6^+^Tim3^−^ progenitor exhausted/stem-like memory phenotype of atherosclerotic aortic T cells is in agreement with a growing body of literature describing progenitor exhausted phenotype of T cells in autoimmune^26,33^ and chronic inflammatory diseases^34^.

Several reports indicated an increased risk for major adverse cardiovascular events in patients receiving ICI therapy, with anti-PD-1 being the most common monotherapy^8–10^. The mechanism behind this increased risk for atherothrombotic events is not fully understood. Alterations in immune cell composition in coronary artery plaques (increased CD3:CD68 ratio) has been shown in patients recently treated with ICI^35^. Here we demonstrate that PD-1 blockade may promote expression of *Cxcl5* in plaques and drive neutrophil accumulation. Further studies are required to determine if neutrophil accumulation is merely a sign of ICI-induced vascular inflammation or whether it plays a role in destabilizing the lesion. Spatial transcriptomic analysis of plaques revealed upregulation of macrophage-related and acute phase-related genes *Saa3*, *Lcn2*, *Mmp3* and *Ccl8*, all of which were previously implicated in driving atherosclerosis or promoting cardiovascular events^36–39^. We did not find any difference in the number of plaque macrophages, but changes in gene expression profile are consistent with activation of myeloid cells in the plaque. Further studies are required to evaluate the interplay between T cells and myeloid cells in the plaque after ICI therapy.

As previously reported^4^, we observed an increase in total aortic T cells after anti-PD-1 treatment in atherosclerotic mice. We report that PD-1 blockade increased IFNγ production by aortic CD4 and CD8 T cells. Furthermore, CD8 T cells in the atherosclerotic aorta of mice treated with anti-PD-1 were heavily skewed toward a PD-1^high^ phenotype and displayed increased levels of PD-1^+^Tim3^+^ expression after treatment with anti-PD-1, suggesting progression toward terminal exhaustion. We also observed formation of lymphocyte foci after PD-1 blockade. Adventitial lymphocyte aggregates of various complexity have been reported in human coronary arteries, where complexity is associated with increasing lesion size and plaque rupture^40^. We propose that the increased local inflammatory response in the plaque caused by anti-PD-1 ICI promotes release of cytokines, leading to generation of advanced adventitial lymphocyte aggregates. This notion is supported by the finding of tertiary lymphoid organ formation in patients with PD-1 blockade-induced myositis^41^. Future studies are needed to discern whether arterial tertiary lymphoid structure formation is accelerated by PD-1 therapy in humans and whether this has implications for cardiovascular pathology. Overall, the association between PD-1 expression and IFNγ production and the responsiveness to anti-PD-1 therapy (PD-1^int^ to PD-1^high^ conversion, generation of TIM3^+^PD-1^+^ terminal exhausted T cells) was more pronounced in CD8 T cells compared to CD4 T cells, implicating PD-1^+^ cytotoxic T cells in ICI-driven atherosclerosis.

Treatment with the non-blocking but depleting anti-PD-1 clone RMP1.30 was used to deplete PD-1^high^ T cells while sparing progenitor exhausted PD-1^int^ T cells. Pre-depletion of PD-1^high^ T cells did not impact responsiveness to PD-1 blockade (using anti-PD-1 clone RMP1.14), suggesting that PD-1^high^ T cells are not required for T cell influx to plaques. Instead, our results highlight Slamf6^+^Tim3^−^PD-1^int^ progenitor exhausted T cells as cellular targets of ICI therapy in atherosclerosis, a finding in agreement with the described role of these cells in cancer and autoimmune diseases^26,42^. However, our findings do not exclude the possibility of other cellular targets of anti-PD-1 therapy. Although not crucial for the accumulation of T cells in plaques, PD-1^high^ T cells, which likely comprise the progeny of PD-1^int^ T cells and de novo induced recently activated T cells, produce IFNγ and may still promote plaque inflammation. Further studies of atherosclerotic mice deficient in either progenitor exhausted or terminally exhausted T cells are required to elucidate their relative contribution to ICI-driven atherosclerosis.

Single-cell RNA-seq of leukocytes in human plaques has revealed the presence of T cells with an exhausted-like gene expression signature^13–15^. We demonstrate that expanded carotid plaque T cells express a diverse transcriptional profile, expressing *TOX*, *LAG3* and *TBX21*, albeit with limited *PDCD1* expression. Further studies are required to validate this finding and explore PD-1 protein expression on locally expanded plaque T cells. Of note, expanded cells found in the plaque may not only be locally proliferating, plaque autoreactive T cells but could also be pathogen-specific T cells that expanded peripherally and then were recruited to the lesion^43^. Our observation of retained functionality in murine aortic T cells expressing PD-1 and Tox warrants caution in inferring functional T cell exhaustion based on RNA expression alone. Several genes associated with exhaustion (such as *TOX* and *PDCD1*) are present not only in functionally exhausted cells but also in polyfunctional memory T cells^44^.

Studies of tumor-infiltrating T cells suggest a linear, non-reversible trajectory from progenitor exhausted T cells to terminally exhausted T cells over time in response to repeated antigen encounter^18^. We found that the majority of PD-1^+^ T cells in plaques displayed a Slamf6^+^Tim3^−^ progenitor exhausted phenotype, retaining cytokine polyfunctionality. The relative scarcity of Slamf6^−^Tim3^+^ terminally exhausted T cells in murine atherosclerotic aortas compared to tumor-infiltrating T cells may be due to several reasons. First, exhausted T cells may have a higher turnover rate in plaques compared to in tumors. Second, the process of generating exhausted T cells may be slower, requiring the study of atherosclerotic mice of a much more advanced age or the use of other non-murine experimental models of atherosclerosis. However, observations in cancer^45^ and chronic infections^46^ counter this argument, as terminal T cell exhaustion was visible already within 1–4 weeks. Third, productive encounters between T cells and antigen-presenting cells in the plaques may be relatively rare due to limited antigen presentation, restricted T cell mobility in plaques or a low frequency of plaque antigen-specific T cells. Of note, we compared aortic T cells with MC38-infiltrating T cells at only one timepoint, and the exhaustion profile is known to change depending on the stage of tumor development^18^ and is likely to differ depending on the type of tumor studied.

Using Nur77–GFP mice, allowing for tracking of recent TCR signaling, we show higher expression of PD-1 in Nur77^+^ T cells, but levels of Nur77 positivity were relatively low in the atherosclerotic aorta. Previous studies estimated that apoB-reactive clones are rare^47^, but whether these clones are enriched in atherosclerotic aortas and how many other clones reactive to plaque antigen exist in parallel is currently unknown. We found that BT3 T cells from human apoB-100 transgenic mice show signs of exhaustion, with high expression levels of PD-1, Tim3 and Tox. Previous studies demonstrated that BT3 T cell transfers limit the development of atherosclerosis by promoting antibody production against LDL, which aids in LDL clearance^24^. It is possible that the upregulation of checkpoint receptors on BT3 T cells, in conjunction with the lipid-lowering effects, contributes to limiting development of atherosclerosis.

We found that levels of circulating PD-1^+^ CD8 T cells in elderly individuals were associated with presence of subclinical atherosclerosis irrespective of classical risk factors such as age, sex, glucose and lipid levels. However, when unadjusted, both levels of PD-1^+^ CD4 T cells and levels of PD-1^+^ CD8 T cells were significantly associated with presence of plaque. In addition, PD-1^+^ CD8 T cells were associated with presence of coronary atherosclerosis after adjusting for age, sex and several cardiovascular risk factors. Of note, analyzed blood samples were taken, on average, 4–8 years after coronary artery imaging; thus, we were unable to consider recent changes in plaque volume. Follow-up analysis of plaque burden in participants in the SCAPIS study is ongoing, which will enable us to evaluate whether levels of PD-1^+^ cells are associated with growth of plaque volume and future cardiovascular events. Previous studies showed that PD-1^+^ T cells are found in healthy adults^48^ and that levels of circulating PD-1-expressing T cells are elevated in patients with autoimmune rheumatoid arthritis^49^. Similarly, in mice, studies showed that PD-1^+^ T cells can promote autoimmune disease^50^ and transplant rejection^27^ and that administration of agonistic anti-PD-1 antibodies limited plaque development in atherosclerotic mice^51^. We demonstrate that PD-1^+^ T cells produce elevated levels of IFNγ upon TCR-driven or cytokine-mediated activation compared to PD-1^−^ counterparts. Increased propensity for cytokine release may mediate the association between levels of these cells and coronary atherosclerosis. Abrogated PD-1-mediated repression by anti-PD-1 therapy may further exacerbate IFNγ release and provide a partial mechanism underlying increased plaque inflammation and other irAEs in patients with cancer. In addition, the observation that PD-1^+^ T cells are responsive to cytokine activation provides an antigen-independent mechanism whereby plaque T cells may be activated and contribute to disease formation. This notion is supported by elegant studies in mice using transgenic T cells specific against plaque-irrelevant antigen that demonstrate that bystander CD8 T cells can accumulate in plaques and respond to innate stimuli (IL-2 and IL-36) by production of IFNγ^52^. Altogether, our findings support a pro-atherogenic role of PD-1-expressing T cells both as mediators of plaque inflammation during the development of atherosclerosis and as cellular targets of PD-1 blocking antibodies, leading to further activation of PD-1^+^ T cells.

With a growing number of patients with cancer benefiting from ICI with long-term survival^53^, amelioration of cardiovascular risk becomes an increasingly relevant clinical priority. Identification of biomarkers of ICI-driven cardiovascular risk is required to pinpoint individuals with a favorable cancer prognosis who would benefit from cardiovigilance or immunomodulatory therapy. Limiting the effects of IFNγ signaling could be a potential strategy to combat ICI-driven cardiovascular disease. However, cautioning against targeting IFNγ signaling itself, treatment of patients with rheumatoid arthritis with the Janus kinase inhibitor tofacitinib led to hyperlipidemia and increased incidence of major adverse cardiovascular events and cancer^54^. IFNγ-inducible chemokines (CXCL9, CXCL10 and CXCL11) that bind to CXCR3 are associated with cardiovascular disease risk in humans^54^, and we observed elevated levels of plaque *Cxcl9* gene expression and increased plasma levels of CXCL9 and CXCL10 after anti-PD-1 therapy. Notably, CXCL9 was previously implicated in ICI-driven myocarditis^55^. However, CXCR3 signaling is necessary for the antitumor efficacy of anti-PD-1 treatment^56^. Modulating downstream effects on myeloid cells may represent a mutually beneficial strategy to combat cardiovascular events after ICI without impairing antitumor efficacy. We recently demonstrated that blockade of IL-1 receptor accessory protein (IL1RAP), the necessary co-receptor for IL-1α/IL-1β, IL-33 and IL-36 signaling, limits plaque inflammation and plaque growth in mice^57^. IL1RAP blockade is currently undergoing clinical development for several cancer indications and would represent an appealing target with potential beneficial effects with regards to both antitumor immunity and plaque inflammation.

Our study has limitations. First, YFP will remain in the cell even after the initial burst of IFNγ production and, thus, may also reflect recent history of IFNγ production in addition to current production. Likewise, Nur77–GFP signal observed in aortic T cells could indicate TCR signaling events occurring in secondary lymphoid organs. Second, plaques develop over longer periods in humans compared to atherosclerotic mice, potentially fostering a different exhaustion profile in human plaque T cells. Future studies are required to elucidate IFNγ-producing PD-1-expressing T cells response to TCR-mediated and innate stimuli in human plaques. Third, analysis of PD-1 on circulating T cells was performed on cells cultured for 24 hours prior to flow cytometric analysis, which could have altered the T cell phenotype. However, we observed a strong correlation between levels of PD-1^+^ cells before and after cell culture, indicating that the culture conditions did not majorly impact our analysis of PD-1. Fourth, human circulating PD-1-expressing T cells comprised T_CM_, T_EM_ and T_EMRA_ subsets, whereas PD-1^−^ T cells comprised a mixture of memory T cells and naive T cells. Further studies are required to assess how cytokine production of PD-1^+^ T cells compares with other non-exhausted effector memory T cell subsets.

In summary, we demonstrate that PD-1 is expressed by aortic IFNγ-producing, progenitor exhausted T cells and that immune checkpoint blockade promotes T cell production of IFNγ in the atherosclerotic aorta. Providing a connection between PD-1 expression and atherosclerosis in humans, we show that levels of PD-1^+^ T cells are associated with presence of subclinical coronary atherosclerosis. Our results demonstrate that PD-1 plays a role in suppressing the activity of pro-inflammatory T cells in atherosclerosis, providing mechanistic insight into the increased cardiovascular risk associated with ICI therapy.

## Methods

### Mice

IFNγ–YFP reporter mice were purchased from The Jackson Laboratory (C.129S4(B6)-Ifng^tm3.1Lky^/J; ‘Great’ mice) and bred with *Apoe*^−*/*−^ mice (The Jackson Laboratory, B6.129P2-Apoe^tmlUnc^/J) in-house to generate homozygous *Ifng*^*YFP/YFP*^*Apoe*^−*/*−^ mice. Nur77–GFP reporter mice were purchased from The Jackson Laboratory (C57BL/6-Tg^(Nr4a1−EGFP/cre)820Khog^/J) and bred with *Apoe*^−*/*−^ mice (The Jackson Laboratory, B6.129P2-Apoe^tmlUnc^/J) in-house to generate *Nur77*^*wt/GFP*^*Apoe*^−*/*−^ mice. To generate double-reporter mice (*Nur77*^*wt/GFP*^*Ifng*^*YFP/YFP*^*Apoe*^−*/*−^), *Ifng*^*YFP/YFP*^*Apoe*^−*/*−^ mice were bred with *Nur77*^*wt/GFP*^*Apoe*^−*/*−^ mice in-house. ApoB-100-reactive TCR transgenic BT3 mice and human *APOB100*-transgenic *Ldlr*^*−/−*^ (HuBL; European Mutant Mouse Archive, 09689) mice were utilized^24^. Euthanization was carried out with intraperitoneal injection of ketamine/xylazine (150 mg kg^−1^, 50 mg kg^−1^) followed by exsanguination via cardiac puncture. Animal experiments were approved by the Malmö/Lund Ethics Committee on Animal Testing at the Lund District Court (ethical permits 8997-18, 11566-2023 and 3112-2020) and in compliance with European Union guidelines (directive 2010/63/EU for the protection of laboratory animals). Mice were randomly assigned into treatment groups when applicable, and cagemates were used as controls. Mice were housed at 22 °C (±2 °C) and 45–65% relative humidity (setpoint 50% relative humidity) in a standard 12-hour light/dark cycle.

### Atherosclerosis experiments

Female and male mice (strains as above), aged 8–11 weeks at the start of the experiment, were fed an HCD (0.21% cholesterol, 21% butter fat; Ssniff, cat. no. E15721-34) for 3–24 weeks depending on the design of the experiment. Refer to individual figures and figure legends for exact duration of each in vivo experiment.

### Tumor transplantation experiment

Murine colon adenocarcinoma MC38 cells (Sigma-Aldrich, SCC172; source: female C57/Bl6 mouse) were cultured in DMEM containing 10% FBS (EmbryoMax; Sigma-Aldrich, ES009-M), 50 μg ml^−1^ gentamicin (Sigma-Aldrich) and 1× penicillin–streptomycin (Sigma-Aldrich). After reaching 80% confluency, cells were harvested with 0.5% trypsin-EDTA (Gibco), washed extensively and resuspended in sterile PBS (Gibco). *Ifng*^*YFP/YFP*^*Apoe*^−*/*−^ mice were implanted with 5 × 10^5^ cells by subcutaneous injection to the right flank. Tumors became palpable after 7 days, and their size (mm^2^) was monitored every 3 days using a digital caliper under anesthesia.

### Tissue preparation and flow cytometry

At euthanization, aorta, blood, plasma, spleen, iliac aortic-draining lymph nodes and hearts were collected. Blood was collected via cardiac puncture with EDTA-coated syringes (0.5 M EDTA; eBioscience). Red blood cells were removed from blood and spleen samples with ammonium–chloride–potassium (ACK) lysis buffer (Thermo Fisher Scientific). Whole aortas were perfused during harvest with PBS and digested by cutting into small pieces and incubating in digestion mix (450 U ml^−1^ Collagenase I, 125 U ml^−1^ Collagenase XI, 60 U ml^−1^ DNAse I, 60 U ml^−1^ hyaluronidase I and 20 mM HEPES buffer) for 1 hour at 37 °C, shaking at 300 r.p.m. Tumor-infiltrating T cells were isolated by cutting the tumor into small pieces and incubating for 1 hour at 37 °C in a digestion mix (Collagenase IV 1 mg ml^−1^, 30 U ml^−1^ DNAse I, 60 U ml^−1^ hyaluronidase I and 20 mM HEPES buffer). ACK lysis buffer (Thermo Fisher Scientific) was used to remove red blood cells. Isolated cells were stained with Zombie Aqua (BioLegend) for live/dead exclusion and incubated with extracellular antibody cocktail. Co-production of T cell cytokines was tested by stimulating splenocytes and pooled aortic digests (*n* = 3–4 per pool) with PMA/ionomycin/brefeldin A (cell stimulation cocktail with brefeldin A; BioLegend) for 4 hours at 37 °C in media (DMEM supplemented with 10% FBS (Gibco)). Staining for IL-2, TNF, IFNγ, Tox and Ki67 was performed after fixing cells in 2% methanol-free formaldehyde (Thermo Fisher Scientific) and permeabilizing cells with pre-made permeabilization buffer (FoxP3/Transcription Factor Staining Buffer Set; eBioscience). Flow cytometry was performed on a Gallios (Beckman Coulter), CytoFLEX (Beckman Coulter) or LSR II (BD Biosciences) flow cytometer. Flow cytometric analysis was performed with FlowJo software version 10.8.0 (Tree Star). Flow cytometry antibodies are detailed in the Supplementary Methods.

### Adoptive transfer of apoB-reactive TCR transgenic CD4 T cells

Splenic CD4 T cells were isolated from male apoB-100-reactive TCR transgenic BT3 mice^24^ or male wild-type (C57Bl/6J) mice using magnetic enrichment (Dynabeads Untouched Mouse CD4 Cells Kit; Invitrogen), followed by intravenous injection of 5 × 10^5^ CD4 T cells to HuBL male mice recipients. HuBL recipient mice were fed an HCD for 3 weeks until euthanization.

### Human carotid plaque single-cell gene expression

Human carotid plaques obtained via carotid endarterectomy were processed as previously described^15^. In brief, plaques were homogenized and digested; CD45^+^ leukocytes were sorted by fluorescence-activated cell sorting (FACS) before single-cell TCR sequencing was performed using 10x Genomics 5′ Single Cell Immune Profiling technology; and clonotypes were defined by TCR amino acid sequencing.

### In vivo IL-2 blockade

Female and male *Ifng*^*YFP/YFP*^*Apoe*^−*/*−^ mice (*n* = 9 per group) were fed an HCD for 10 weeks, administering three injections of isotype IgG2a or anti-IL-2 (clone JES6-1, 0.5 mg per injection) 5 days apart during the last 2 weeks of the experiment.

### Anti-PD-1 treatments

Long-term PD-1 blockade (6 weeks of treatment). Female and male *Ifng*^*YFP/YFP*^*Apoe*^−*/*−^ mice were fed an HCD for 10 weeks before starting injections. Mice were randomly assigned to receive intraperitoneal injections of either murinized and effector-less anti-PD-1 antibody (clone: RMP1-14, anti-mPD-1-mIgG1e3; InvivoGen, mpd1-mab15-50) or isotype control IgG2a (anti-mPD-1-mIgG1e3, InvivoFit; InvivoGen) biweekly for 6 weeks at 10 mg kg^−1^ (*n* = 12 per group).

Short-term PD-1 blockade (3 weeks of treatment). Female and male *Ifng*^YFP/YFP^*Apoe*^*−/−*^mice were fed an HCD for 21 weeks before starting injections. Mice were randomly assigned to receive intraperitoneal injections of either anti-PD-1 antibody (clone: RMP1-14; Bio X Cell) or isotype control IgG2a (anti-trintrophenol, clone: 2A3; Bio X Cell) biweekly for 3 weeks at 10 mg kg^−1^ (*n* = 11–12 per group).

PD-1 blockade in tumor-bearing mice. Female and male *Ifng*^*YFP/YFP*^*Apoe*^−*/*−^ mice (*n* = 7 per group) were fed an HCD for 10 weeks before injection of PBS or MC38 cells (2 × 10^5^ cells) and treated with biweekly injections of anti-PD-1 (clone: RMP1-14, 10 mg kg^−1^; Bio X Cell) or IgG2a isotype control (anti-trintrophenol, clone: 2A3; Bio X Cell), starting 1 week after tumor implantation. Mice were monitored for tumor growth, and tumors and hearts were harvested 4 weeks after tumor implantation at euthanization.

### Combination of PD-1 depletion and PD-1 blockade

Female and male *Ifng*^*YFP/YFP*^*Apoe*^−*/*−^ mice (*n* = 10–13 per group) were fed an HCD for 4 months and treated with (1) rat IgG2b isotype control (in vivo GOLD Functional Grade, Leinco Technologies, 0.5 mg per injection) for 3 weeks; (2) depleting anti-PD-1 (clone: RMP1.30, rat IgG2b, in vivo GOLD Functional Grade, Leinco Technologies, 0.5 mg per injection) for 3 weeks; (3) rat IgG2b isotype control (in vivo GOLD Functional Grade, Leinco Technologies, 0.5 mg per injection) for 1 week followed by anti-PD-1 (clone: RMP1.14, 10 mg kg^−1^; Bio X Cell) for 2 weeks; or (4) depleting anti-PD-1 (clone: RMP1.30, rat IgG2b, in vivo GOLD Functional Grade, Leinco Technologies, 0.5 mg per injection) for 1 week prior to biweekly injections of anti-PD-1 (clone: RMP1.14, 10 mg kg^−1^; Bio X Cell) for 2 weeks. For this experiment, hearts were formalin fixed and paraffin embedded (FFPE) prior to sectioning and staining.

### Histology and immunofluorescence

Hearts were snap frozen with liquid nitrogen upon harvest and mounted in Optimal Cutting Temperature (OCT) compound (VWR), and aortic root cross-sections were collected starting from the identification of the aortic valve at 6-μm thickness. To determine plaque size, aortic root cross-sections were stained with Oil Red O and counterstained with Harris’ hematoxylin. For immunohistochemical staining, aortic root cross-sections were stained with the following: anti-CD3 (Armenian hamster anti-mouse, BioLegend) as primary antibody, biotinylated goat anti-hamster (Vector Laboratories) as secondary antibody and Armenian hamster IgG2a (BioLegend) as isotype control; anti-PD-L1 (rat anti-mouse, BioLegend) as primary antibody, biotinylated rabbit anti-rat (Vector Laboratories) as secondary antibody and rat IgG2b (Abcam) as isotype control; anti-CD19 antibody (rabbit anti-mouse, Abcam), biotinylated anti-rabbit IgG as secondary antibody and rabbit IgG as isotype control; anti-Ly6G antibody (clone: 1A8, rat anti-mouse IgG, BD Biosciences) as primary antibody, biotinylated rabbit anti-rat (Vector Laboratories) as secondary antibody and rat IgG2a as isotype control; and anti-CD68 (clone: FA-11, rat anti-mouse IgG, Bio-Rad) as primary antibody, biotinylated rabbit anti-rat (Vector Laboratories) as secondary antibody and rat IgG2a as isotype control. For FFPE hearts, antigen retrieval was performed using sodium citrate buffer (pH 6) before continuing with primary antibody staining (anti-CD3, clone:SP7, Abcam) followed by ImmPRESS HRP goat anti-rabbit (Vector Laboratories). After antibody staining, sections were incubated in ABC Elite (Vector Laboratories), developed with DAB ImmPACT kit (Vector Laboratories) and counterstained with Mayer’s hematoxylin. For co-localization immunofluorescent staining of T cells and B cells, aortic root cross-sections were stained with the same primary antibodies for CD3 and CD19 as above, and secondary antibodies were Alexa Fluor 555-conjugated (Invitrogen) for CD3 identification and Alexa Fluor 488-conjugated (Invitrogen) for CD19 identification. After antibody staining, sections were incubated in Sudan Black (0.03%) and counterstained with DAPI and imaged by confocal microscopy.

### scRNA-seq and spatial transcriptomics

Gene expression analysis of CD44^+^PD-1^+/−^ T cells was performed using a previously described scRNA-seq dataset^58^. Spatial transcriptomic analysis of aortic root plaques from isotype IgG-treated (*n* = 1) or anti-PD-1-treated (*n* = 1) mice was performed using the 10x Visium (10x Genomics) platform. Cross-sections of FFPE murine hearts with 5-μm thickness were prepared according to the manufacturer’s instructions. Loupe Browser was used to visualize and select the tissue region of interest (subvalvular plaques). Differential gene expression analysis and visualization were performed in R (version 4.x) using the tidyverse package suite (dplyr and ggplot2). *P* values were adjusted for multiple comparisons using the Benjamini–Hochberg method to control for the false discovery rate (FDR). Genes with an adjusted *P* < 0.05 and log_2_ fold change > 1 were considered significantly differentially expressed.

### PBMCs and coronary artery bioimaging

PBMCs were collected from individuals participating in the Functional IMmunity and CardiOvascular Disease (FIMCOD) substudy between January and June 2022. The study was approved by the Swedish Ethical Review Authority (permit 2021-04001), and all participants gave written informed consent.

The FIMCOD substudy consists of men and women aged 65–72 years who had previously participated in the SCAPIS study (https://www.scapis.org/) in Malmö, Sweden. SCAPIS is a general population-based prospective study that, between 2014 and 2018, analyzed cardiovascular and pulmonary health in recruited women and men aged 50–64 years^59^. Inclusion criteria for the recruitment of patients to the FIMCOD study from SCAPIS were individuals (1) aged 65 years or older, (2) having been vaccinated against COVID-19 (three doses) and (3) without a history of self-reported COVID-19 disease or COVID-19 disease of any person in the same household. Metabolic profile (plasma levels of LDL, HDL, triglycerides and glucose) was measured by clinical chemistry analysis (Skåne University Hospital) contemporaneously to PBMC collection in the FIMCOD study.

Between 2014 and 2018, study participants were analyzed by CCTA to assess the degree of coronary atherosclerosis, as previously described^60^. SIS, the total number of coronary segments with atherosclerosis irrespective of the degree of stenosis, was used to characterize overall coronary plaque burden (no coronary atherosclerosis, SIS = 0) or presence of any degree of coronary atherosclerosis (SIS ≥ 1). Study participants were also assigned a modified version of the Duke score, grading individuals based on proximal location and stenosis of coronary artery atherosclerosis on a seven-grade scale^28^.

### Flow cytometry of human PBMCs

Frozen PBMCs (*n* = 675, SCAPIS/FIMCOD cohort) were thawed and cultured in complete RPMI (cRPMI; 10% FBS, penicillin–streptomycin, L-glutamine, sodium pyruvate and non-essential amino acids) in the presence of anti-CD49d/anti-CD28 (BD Biosciences, cat. no. 347690) for 24 hours. Cells were washed, stained with Zombie Aqua (BioLegend) and incubated with extracellular antibody cocktail (see Supplementary Methods). Samples were acquired on a Gallios flow cytometer and analyzed by FlowJo software.

### In vitro cytokine stimulation of human PBMCs

Frozen PBMCs (*n* = 15, SCAPIS/FIMCOD cohort) were thawed and resuspended in cRPMI media. Cells were split into three conditions: cRPMI alone, TCR stimulation and cytokine stimulation. For TCR stimulation, cells were transferred to anti-CD3 (2 μg ml^−1^, clone OKT3)-coated wells and supplemented with anti-CD49d/anti-CD28 co-stimulation (BD Biosciences). For cytokine stimulation, cells were transferred to uncoated wells and 50 ng ml^−1^ IL-12p70 (PeproTech, cat. no. 200-12H-2UG), 50 ng ml^−1^ IL-15 (PeproTech, cat. no. 200-15-2UG) and 250 ng ml^−1^ IL-18 (R&D Systems, cat. no. 9124-IL-010, with carrier). Cells were stimulated for a total of 24 hours, and brefeldin A was added to all wells 5 hours before cells were harvested. Cells were washed and stained with Zombie Aqua (BioLegend) and extracellular antibodies, followed by cell fixation and permeabilization (Invitrogen, FIX & PERM Cell Permeabilization Kit) and intracellular staining for IFNγ.

### Statistics

All data (excluding SIS correlation and tissue comparisons) were tested for normal distribution (Kolmogorov–Smirnov normality test) and analyzed with unpaired two-tailed Student’s *t*-test, Mann–Whitney *U*-test, one-way ANOVA or Kruskal–Wallis test, accordingly, using GraphPad Prism version 10.4.0 (GraphPad Software). Data comparing different tissues within the same mice (Figs. 1f,g and 2 and Extended Data Fig. 2) were analyzed with Wilcoxon matched-pairs test. To test association of PD-1 expression on circulating T cells in humans with coronary atherosclerosis, binary logistic regression analysis (for presence of plaque) or linear regression analysis (for Duke score) adjusted for risk factors was performed using SPSS software (version 27). A *P* value of less than 0.05 was considered significant, and *P* values less than 0.10 are reported. All statistical tests were performed two-sided unless stated otherwise in the figure legend. Multiple comparison adjustments, if performed, are stated in the corresponding legend.

### Reporting summary

Further information on research design is available in the Nature Portfolio Reporting Summary linked to this article.

## Supplementary information

Supplementary Information Flow cytometry antibodies.

Reporting Summary

Supplementary Tables 1 and 2 Supplementary Table 1: scRNA-seq data comparing gene expression of PD-1^+^ compared to PD-1^−^ aortic T cells from atherosclerotic *Ldlr*^−*/*−^ mice. Supplementary Table 2: Spatial transcriptomic gene expression analysis comparing atherosclerotic plaques from *Ifng*^*YFP/YFP*^*Apoe*^−*/*−^ mice treated with anti-PD-1 antibodies or control isotype IgG.

## Source data

Source Data Figs. 1–8 Statistical Source Data.

Source Data Extended Data Figs. 1–8 Statistical Source Data.

## Data Availability

Aortic scRNA-seq analysis of plaque T cells from *Ldlr*^−*/*−^ mice^16^ is included in Supplementary Table 1. Data availability of matched PBMC and plaque T cell clonality single-cell TCRβ RNA sequencing is described fully in Depuydt et al.^15^. Visium spatial transcriptomics data are available at OSF (10.17605/OSF.IO/QR2VP). All other source data (flow cytometric and histological) presented in this study can be located in the provided Source Data files. Study participant data related to PD-1 measurement of PBMCs in the SCAPIS cohort cannot be made openly available due to the sensitive nature of the personal data. Requests for data, analytical methods or study materials should be directed to the corresponding author or the study organization (https://www.scapis.org/). Access will be granted only if the request complies with Swedish legislation.
