# Supplementary material for: Progenitor exhausted PD-1+ T cells are cellular targets of immune checkpoint inhibition in atherosclerosis
Source: Nat Cardiovasc Res. 2025 Oct 7;4(10):1311–28. doi: 10.1038/s44161-025-00713-2 (PMC12520982; doi:10.1038/s44161-025-00713-2)
Supplement: Supplementary file 1 — Flow cytometry antibodies. [file 44161_2025_713_MOESM1_ESM.pdf]

nature cardiovascular research

Supplementary information https://doi.org/10.1038/s44161-025-00713-2

Progenitor exhausted PD-1^{+} T cells are cellular targets of immune checkpoint inhibition in atherosclerosis

In the format provided by the authors and unedited

Mouse flow cytometry antibodies

Antibody	Clone	Dilution	Supplier
CD3-BV510	145-2C11	1:200	BioLegend
CD4-APC/Cy7	GK1.5	1:100	BioLegend
CD4-BV605	RM4.5	1:1000	BioLegend
CD8-AF700	53-6.7	1:100	BioLegend
CD8-BV750	53-6.7	1:1000	BD BioSciences
CD44-PerCP/Cy5.5	IM7	1:100	BioLegend
CD45-BV421	30-F11	1:100	BioLegend
CD45.1-APC	A20	1:200	eBioscience
CD45.2-BV605	104	1:100	BioLegend
CD69-APC	H1.2F3	1:100	BioLegend
CD103-PE	2E7	1:100	BioLegend
CXCR5-BV605 (CD185)	L138D7	1:100	BioLegend
IFNγ-APC	XMG1.2	1:100	BioLegend
IFNγ-AF700	XMG1.2	1:100	BioLegend
IL2-PE	JES6-5H4	1:100	BioLegend
IL7Rα-APC (CD127)	A7R34	1:100	BioLegend
Ki67-APC	16A8	1:100	BioLegend
KLRG1-PE	2F1/KLRG1	1:100	BioLegend
LAG3-BV650 (CD223)	C9B7W	1:100	BioLegend
Ly6C-eFluor450	HK1.4	1:100	Invitrogen
PD1-PE/Dazzle 594 (CD279)	29F.1A12	1:100	BioLegend
PD1-BV421 (CD279)	29F.1A12	1:100	BioLegend
PSGL1-BV786 (CD162)	2PH1	1:100	BD Biosciences
Slamf6-PE (Ly-108)	eBio13G3-19D	1:200	Invitrogen
TCRβ-PE/Cy7	H57-597	1:100	BioLegend
Tim3-APC (CD366)	RMT3-23	1:100	BioLegend
Tim3-PE/Cy7	RMT3-23	1:200	BioLegend
TNFα-BV421	MP6-XT22	1:100	BioLegend
Tox-PE	REA473	1:100	Miltenyi BioTec
Tox-eFluor660	TXRX10	1:200	eBioscience
TRBv31-FITC	14-2	1:1000	BD BioSciences
CD16/32	93	1:100	BioLegend

Human flow cytometry antibodies

Antibody	Clone	Dilution	Supplier
CCR7-PE/Dazzle 594	G043H7	1:25	BioLegend
CD3-APC/Cy7	HIT13a	1:100	BioLegend
CD4-AF700	RPA-T4	1:200	BioLegend
CD8-PerCP/Cy5.5	RPA-T8	1:40	BioLegend
CD45RA-AF488	HI100	1:80	BioLegend
CD45RO-AF488	UCHL1	1:40	BioLegend
IFNγ-APC	4S.B3	1:40	BioLegend
PD1-BV421	EH12.2H7	1:40	BioLegend
TruStain FcX	HIT13a	1:100	BioLegend
