# Supplementary material for: Progenitor exhausted PD-1+ T cells are cellular targets of immune checkpoint inhibition in atherosclerosis
Source: Nat Cardiovasc Res. 2025 Oct 7;4(10):1311–28. doi: 10.1038/s44161-025-00713-2 (PMC12520982; doi:10.1038/s44161-025-00713-2)
Supplement: Supplementary file 2 — Reporting Summary [file 44161_2025_713_MOESM2_ESM.pdf]

nature portfolio

Corresponding author(s): Daniel Engelbertsen, Megan Mulholland

Last updated by author(s): Aug 1, 2025

Reporting Summary

Nature Portfolio wishes to improve the reproducibility of the work that we publish. This form provides structure for consistency and transparency in reporting. For further information on Nature Portfolio policies, see our Editorial Policies and the Editorial Policy Checklist.

Statistics

For all statistical analyses, confirm that the following items are present in the figure legend, table legend, main text, or Methods section.

n/a	Confirmed	
☐	☒	The exact sample size (*n*) for each experimental group/condition, given as a discrete number and unit of measurement
☐	☒	A statement on whether measurements were taken from distinct samples or whether the same sample was measured repeatedly
☐	☒	The statistical test(s) used AND whether they are one- or two-sided<br>*Only common tests should be described solely by name; describe more complex techniques in the Methods section.*
☐	☒	A description of all covariates tested
☐	☒	A description of any assumptions or corrections, such as tests of normality and adjustment for multiple comparisons
☐	☒	A full description of the statistical parameters including central tendency (e.g. means) or other basic estimates (e.g. regression coefficient) AND variation (e.g. standard deviation) or associated estimates of uncertainty (e.g. confidence intervals)
☐	☒	For null hypothesis testing, the test statistic (e.g. *F*, *t*, *r*) with confidence intervals, effect sizes, degrees of freedom and *P* value noted<br>*Give P values as exact values whenever suitable.*
☒	☐	For Bayesian analysis, information on the choice of priors and Markov chain Monte Carlo settings
☒	☐	For hierarchical and complex designs, identification of the appropriate level for tests and full reporting of outcomes
☒	☐	Estimates of effect sizes (e.g. Cohen's *d*, Pearson's *r*), indicating how they were calculated

Our web collection on statistics for biologists contains articles on many of the points above.

Software and code

Policy information about availability of computer code

Data collection	No software was used for data collection.
Data analysis	QuPath software, v0.2.3, (https://doi.org/10.1038/s41598-017-17204-5) was used to analyze immunohistochemistry. R (v4.x) was used to analyze differential gene expression of sequencing data utilizing the tidyverse package suite (dplyr and ggplot2). Flow cytometry data was analyzed with FlowJo software v10.8.0 (Tree Star Inc., OR, USA). Statistical analysis was completed in GraphPad Prism (v10.4.0) and SPSS (IBM, v27) software.

For manuscripts utilizing custom algorithms or software that are central to the research but not yet described in published literature, software must be made available to editors and reviewers. We strongly encourage code deposition in a community repository (e.g. GitHub). See the Nature Portfolio guidelines for submitting code & software for further information.

Data

Policy information about availability of data

All manuscripts must include a data availability statement. This statement should provide the following information, where applicable:

- Accession codes, unique identifiers, or web links for publicly available datasets
- A description of any restrictions on data availability
- For clinical datasets or third party data, please ensure that the statement adheres to our policy

Study participant data related to PD1 measurement of peripheral blood mononuclear cells in the SCAPIS cohort, cannot be made openly available due to the

sensitive nature of the personal data. Contacting the corresponding author or study organization (www.scapis.org), procedures for sharing data, analytic methods, and study materials can be arranged when aligned with Swedish legislation. The raw scTCR-seq data from the Athero-Express cohort are not publicly available due to research participant privacy/consent. These data and the bulk TCRβ sequencing data can be accessed via DataverseNL at this address: https://doi.org/10.34894/DDYKLL. There are restrictions on use by commercial parties and on sharing openly based on (inter)national laws and regulations and written informed consent. Therefore, these data (and additional clinical data) are available only upon discussion and signing a data sharing agreement (see Terms of Access in DataverseNL) and within a specially designed UMCU-provided environment. Aortic single-cell RNA sequencing analysis of plaque T cells from Ldlr-/- mice (Smit, V et al.) is included in Supplementary Table 1. Spatial transcriptomic analysis from IfngYFP/YFPApoe-/- mice treated with anti-PD1 antibodies or isotype IgG is included in Supplementary Table 2. All other source data (flow cytometric, histological) presented in this study can be located in the provided Source Data files.

Research involving human participants, their data, or biological material

Policy information about studies with human participants or human data. See also policy information about sex, gender (identity/presentation), and sexual orientation and race, ethnicity and racism.

Reporting on sex and gender	The SCAPIS study is a general population-based prospective study that invited both men and women to participate without any bias. Individuals recruited in our follow-up study were not selected based on biological sex. The percentage of female participants is detailed in Extended Data Figure 8A.
Reporting on race, ethnicity, or other socially relevant groupings	We do not have access to any data related to ethnicity or race.<br>However, our study was performed at a single study centre in Sweden, potentially limiting the application of our results to patient populations of other sociodemographic groups.
Population characteristics	Study participants of the SCAPIS study, residing in the city of Malmö, Sweden, were recruited from the general population. Within this group, we invited individuals over the age of 65 that had received three doses of COVID19 vaccine without a history of COVID19 infection to participate in our follow-up study ("Functional IMmunity and CardiOvascular Disease" or FIMCOD). The rationale for these selection criteria is that a primary aim of the FIMCOD study is to evaluate the relationship between vaccine responsiveness and cardiovascular disease. Results from these vaccine studies are not part of the present investigation.<br><br>A full report describing the population characteristics and details of the FIMCOD study is currently under consideration for publication (Andersson et al.). Briefly, the study population displayed: 12.5% history of cardiovascular disease, 9.2% history of diabetes, 69.5% presence of carotid plaque, and 8.2% smoking.
Recruitment	Study participants that had previously participated in the SCAPIS study were recruited to participate in the follow-up study (FIMCOD) where we collected blood and plasma (collected Q1-Q2 2022). SCAPIS participants received a letter describing the study and were also contacted by telephone by the Lund University Clinical Research Unit. All participants provided informed consent.
Ethics oversight	The protocol was approved by the The Swedish Ethical Review Authority (#2021-04001).

Note that full information on the approval of the study protocol must also be provided in the manuscript.

Field-specific reporting

Please select the one below that is the best fit for your research. If you are not sure, read the appropriate sections before making your selection.

☒ Life sciences ☐ Behavioural & social sciences ☐ Ecological, evolutionary & environmental sciences

For a reference copy of the document with all sections, see nature.com/documents/nr-reporting-summary-flat.pdf

Life sciences study design

All studies must disclose on these points even when the disclosure is negative.

Sample size	No prior sample size calculations were performed. Number of mice in each experiment are within conventional sample sizes used in the field, taking into account the different levels of variability between analytical methods (e.g. histology versus flow cytometry versus gene expression analysis).
Data exclusions	In Extended data Fig. 5, a lymph node sample was excluded due to low quality flow cytometry. Two samples were excluded from aortic flow cytometry analysis in Extended Data Fig. 6 due to technical error. In Figure 7B-C, 4 data points from day 0 blood PD1 kinetics and 3 data points from day 7 blood kinetics were removed due to insufficient quantity of blood taken during the blood draw (data points were mixed between different mice on different days and within both treatment groups).
Replication	In all mouse experiments, the same gating strategy was used to identify PD1 populations and consistently yielded similar results. In vitro simulation of human PBMCs was performed twice (n=7, n=8) and the two separate experiments were pooled during data analysis, as shown in Figure 7 and Extended Data Figure 7 (n=15).
Randomization	For anti-PD1 experiment, mice were randomized into treatment groups via computer-generated randomization before any encounters with the mice and cage-mates were used as controls. The same computer-generated randomization was used when selecting PBMC samples to use in the in vitro stimulation experiment.

Blinding	Mice were assigned psuedomized codes during harvest and data acquitision and analysis. Unmasking was performed only at the final step of statistical analysis and figure construction.

Behavioural & social sciences study design

All studies must disclose on these points even when the disclosure is negative.

Study description	*Briefly describe the study type including whether data are quantitative, qualitative, or mixed-methods (e.g. qualitative cross-sectional, quantitative experimental, mixed-methods case study).*
Research sample	*State the research sample (e.g. Harvard university undergraduates, villagers in rural India) and provide relevant demographic information (e.g. age, sex) and indicate whether the sample is representative. Provide a rationale for the study sample chosen. For studies involving existing datasets, please describe the dataset and source.*
Sampling strategy	*Describe the sampling procedure (e.g. random, snowball, stratified, convenience). Describe the statistical methods that were used to predetermine sample size OR if no sample-size calculation was performed, describe how sample sizes were chosen and provide a rationale for why these sample sizes are sufficient. For qualitative data, please indicate whether data saturation was considered, and what criteria were used to decide that no further sampling was needed.*
Data collection	*Provide details about the data collection procedure, including the instruments or devices used to record the data (e.g. pen and paper, computer, eye tracker, video or audio equipment) whether anyone was present besides the participant(s) and the researcher, and whether the researcher was blind to experimental condition and/or the study hypothesis during data collection.*
Timing	*Indicate the start and stop dates of data collection. If there is a gap between collection periods, state the dates for each sample cohort.*
Data exclusions	*If no data were excluded from the analyses, state so OR if data were excluded, provide the exact number of exclusions and the rationale behind them, indicating whether exclusion criteria were pre-established.*
Non-participation	*State how many participants dropped out/declined participation and the reason(s) given OR provide response rate OR state that no participants dropped out/declined participation.*
Randomization	*If participants were not allocated into experimental groups, state so OR describe how participants were allocated to groups, and if allocation was not random, describe how covariates were controlled.*

Ecological, evolutionary & environmental sciences study design

All studies must disclose on these points even when the disclosure is negative.

Study description	*Briefly describe the study. For quantitative data include treatment factors and interactions, design structure (e.g. factorial, nested, hierarchical), nature and number of experimental units and replicates.*
Research sample	*Describe the research sample (e.g. a group of tagged Passer domesticus, all Stenocereus thurberi within Organ Pipe Cactus National Monument), and provide a rationale for the sample choice. When relevant, describe the organism taxa, source, sex, age range and any manipulations. State what population the sample is meant to represent when applicable. For studies involving existing datasets, describe the data and its source.*
Sampling strategy	*Note the sampling procedure. Describe the statistical methods that were used to predetermine sample size OR if no sample-size calculation was performed, describe how sample sizes were chosen and provide a rationale for why these sample sizes are sufficient.*
Data collection	*Describe the data collection procedure, including who recorded the data and how.*
Timing and spatial scale	*Indicate the start and stop dates of data collection, noting the frequency and periodicity of sampling and providing a rationale for these choices. If there is a gap between collection periods, state the dates for each sample cohort. Specify the spatial scale from which the data are taken*
Data exclusions	*If no data were excluded from the analyses, state so OR if data were excluded, describe the exclusions and the rationale behind them, indicating whether exclusion criteria were pre-established.*
Reproducibility	*Describe the measures taken to verify the reproducibility of experimental findings. For each experiment, note whether any attempts to repeat the experiment failed OR state that all attempts to repeat the experiment were successful.*
Randomization	*Describe how samples/organisms/participants were allocated into groups. If allocation was not random, describe how covariates were controlled. If this is not relevant to your study, explain why.*
Blinding	*Describe the extent of blinding used during data acquisition and analysis. If blinding was not possible, describe why OR explain why blinding was not relevant to your study.*

Did the study involve field work? ☐ Yes ☒ No

Reporting for specific materials, systems and methods

We require information from authors about some types of materials, experimental systems and methods used in many studies. Here, indicate whether each material, system or method listed is relevant to your study. If you are not sure if a list item applies to your research, read the appropriate section before selecting a response.

Materials & experimental systems

n/a	Involved in the study
☐	☒ Antibodies
☐	☒ Eukaryotic cell lines
☒	☐ Palaeontology and archaeology
☐	☒ Animals and other organisms
☒	☐ Clinical data
☒	☐ Dual use research of concern
☒	☐ Plants

Methods

n/a	Involved in the study
☒	☐ ChIP-seq
☐	☒ Flow cytometry
☒	☐ MRI-based neuroimaging

Antibodies

Antibodies used

Antibodies used in vivo can be found within the Methods section of the paper. In summary: rat anti-mouse PD1 (clone: RMP1-14, cat: BE0146; dosed 10mg/kg) and isotype control rat IgG2a (anti-trintrophenol, clone: 2A3, cat: BE0089; dosed 10mg/kg) purchased from BioXcell for short-term PD1 blockade experiments; mouse anti-mouse PD1 (clone: RMP1-14, anti-mPD-1-mIgG1e3, cat: mpd1-mab15-50; dosed 10mg/kg) and isotype control mouse IgG2a (Anti-β-Gal-mIgG1e3, cat: bgal-mab15-02; dosed 10mg/kg) purchased from InVivoGen for long-term PD1 blockade experiments; rat anti-mouse PD1 (clone: RMP1-30, cat: C3442; dosed 0.5mg/injection) and isotype control rat IgG2b (clone: 1-2, cat: I-1034; dosed 0.5mg/injection) purchased from Leinco Technologies for PD1 depletion experiments; rat anti-mouse IL2 (clone: JES6-1A12, cat: BE0043; dosed 0.5mg/injection) and isotype control rat IgG2a (anti-trintrophenol, clone: 2A3, cat: BE0089; dosed 0.5mg/injection) purchased from BioXcell for IL2 blockade experiments.

A full list of antibodies (with dilutions) used for flow cytometry experiments is included in the Supplemental Methods document.

Primary and secondary antibodies used in immunohistochemistry (IHC) and immunofluorescence (IF) can be found within the Methods section of the paper. In summary: CD3 IHC (primary: Armenian hamster anti-mouse, 100302, BioLegend; secondary: biotinylated goat anti-hamster, BA-9100, Vector Laboratories; isotype control: Armenian hamster IgG2a, 400939, BioLegend); PDL1 IHC (primary: rat anti-mouse, 124302, BioLegend; secondary: biotinylated rabbit anti-rat, BA-4001, Vector Laboratories; isotype control: rat IgG2b, 18539, Abcam); CD19 IHC (primary: rabbit anti-mouse, EPR23174-145, Abcam; secondary: biotinylated goat anti-rabbit IgG, BA1000, Vector Laboratories; isotype control: rabbit IgG polyclonal, ab27478, Abcam); CD3 IF (primary: Armenian hamster anti-mouse, 100302, BioLegend; secondary: AF555-conjugated goat anti-hamster, A78964, Invitrogen; isotype control: Armenian hamster IgG2a, 400939, BioLegend); CD19 IF: (primary: rabbit anti-mouse, EPR23174-145, Abcam; secondary: AF488-conjugated goat anti-rabbit, ab150077, Abcam; isotype control: rabbit IgG polyclonal, ab27478, Abcam).

Validation

All antibodies underwent significant quality control procedures by the manufacturers.

As stated on their website (https://bioxcell.com/educational-articles/quality-control), InVivoMAb from BioXCell are subjected to rigorous quality control testing, including screening for binding specificity against antigen and negative control antigen (immunoblot), ensuring monomer content of ≥95% (size exclusion chromatography), and isotype confirmation (rapid lateral flow antibody isotyping assay; testing host species, isotype class, subtype class, light-chain identity) to ensure lot-to-lot consistency of antibodies.

BioLegend has a similar statement and commitment to rigorous quality control on their website (https://www.biolegend.com/en-us/quality/quality-control). Specificity of their flow cytometry antibodies is tested in 1-3 target cell types along with positive and negative cell types (single- or multi-color analysis). Once specificity is confirmed, each new lot must perform with similar intensity to the in-date reference lot. Brightness (MFI) is evaluated from both positive and negative populations. Each lot product is validated by QC testing with a series of titration dilutions. Antibodies from BioLegend used in IHC and IF (https://www.biolegend.com/en-us/quality/quality-control) are tested for purity by SDS-PAGE gel electrophoresis (IgG antibodies are required to have purity >95%). Fluorophore and enzyme-conjugated antibodies follow strict manufacturing specifications to ensure performance, and each lot is validated by QC testing as stated on the TDS to confirm specificity and lot-to-lot consistency.

Abcam's website (https://go.myabcam.com/BiophysicalQuality) states its use of biophysical QC to detects any impurities and aggregates in their antibodies, generating data to validate subsequent batches, guaranteeing the highest specificity, sensitivity and consistency every time. This approached utilizes various techniques, including liquid chromatography-mass spectrometry (LC-MS), dynamic light scattering (DLS), and high-performance liquid chromatography (HPLC) to assess sequence identity, sequence integrity, aggregation, purity, and concentration.

Eukaryotic cell lines

Policy information about cell lines and Sex and Gender in Research

Cell line source(s)	The MC38 adenocarcinoma cell line was purchased from Sigma-Aldrich (cat# SCC172). This cell line derived from a female C57Bl/6 mouse.
Authentication	MC38 cell line was not authenticated in-house. The manufacturer verified the cell line to be of mouse origin and negative for human, rat, Chinese hamster, non-human primate and golden Syrian hamster interspecies contamination as assessed by a contamination-clear panel by Charles River Animal Diagnostic Service.
Mycoplasma contamination	MC38 cell line was not tested in-house for mycoplasma contamination. However, the manufacturer certifies that the cell line tested negative for mycoplasma contamination before shipping as well as negative for infectious disease against a Mouse Essential CLEAR panel performed by Charles River Animal Diagnostic Service.
Commonly misidentified lines (See ICLAC register)	No commonly misidentified cell lines were used in this study.

Animals and other research organisms

Policy information about studies involving animals; ARRIVE guidelines recommended for reporting animal research, and Sex and Gender in Research

Laboratory animals	IFN-γ-YFP reporter mice (Jackson Laboratory; strain C.129S4(B6)-Ifngtm3.1Lky/J, “Great” mice) were bred with Apoe-/- (Jackson Laboratory; strain B6.129P2-ApoetmlUnc/J) in-house to generate homozygous Ifng(YFP/YFP)Apoe(-/-) mice. Nur77-GFP reporter mice (Jackson Laboratory; strain C57BL/6-Tg(Nr4a1-EGFP/cre)820Khog/J) were bred with Apoe-/- (Jackson Laboratory, (B6.129P2-ApoetmlUnc/J) in-house to generate Nur77(wt/GFP)Apoe(-/-) mice. To generate double-reporter mice (Nur77wt/GFPIfngYFP/YFPApoe-/-), IfngYFP/YFPApoe-/- mice were bred with Nur77wt/GFPApoe-/- mice in-house. ApoB100-reactive TCR transgenic BT3 mice and human APOB100-transgenic Ldlr−/− (HuBL, European mutant mouse archive 09689) were generated as previously described in reference 16.<br><br>Female and male mice (strains as above), aged 8-11 weeks at the start of experiment, were fed a high cholesterol diet (HCD; 0.21% cholesterol, 21% butter fat, cat# E15721-34, Ssniff) for 3-24 weeks depending on the design of the experiment. Refer to individual figures and figure legends for exact duration of each in vivo experiment.<br><br>Mice were housed at Lund University CMU and CRC animal facilities, which are kept at 22 degrees C (+/- 2 degrees) with a standard 12-hour light/12-hour dark cycle of and humidity is maintained in individual cages via HVAC between 45-65% relative humidity (setpoint 50% relative humidity).
Wild animals	The study did not include wild animals.
Reporting on sex	Both female and male mice were utilized in all mouse experiments excluding HuBL-BT3 transfer experiments, in which one sex (male) was used due to nature of adoptive transfer experiments. No sex-based analysis was performed as this is outside the scope of this study. Excluding HuBL mice experiment, the study included 47% female mice and 53% male mice. Including HuBL mice experiment the study included 46% female mice and 54% male mice.
Field-collected samples	The study did not involve field-collected samples.
Ethics oversight	All animal experiments were approved by local ethics committee (Malmö/Lund Ethics Committee on Animal Testing at the Lund District Court; ethical permits: #8997-18, #11566-2023 and #3112-2020) and in compliance with EU guidelines (directive 2010/63/EU for the protection of laboratory animals).

Note that full information on the approval of the study protocol must also be provided in the manuscript.

Plants

Seed stocks	*Report on the source of all seed stocks or other plant material used. If applicable, state the seed stock centre and catalogue number. If plant specimens were collected from the field, describe the collection location, date and sampling procedures.*
Novel plant genotypes	*Describe the methods by which all novel plant genotypes were produced. This includes those generated by transgenic approaches, gene editing, chemical/radiation-based mutagenesis and hybridization. For transgenic lines, describe the transformation method, the number of independent lines analyzed and the generation upon which experiments were performed. For gene-edited lines, describe the editor used, the endogenous sequence targeted for editing, the targeting guide RNA sequence (if applicable) and how the editor was applied.*
Authentication	*Describe any authentication procedures for each seed stock used or novel genotype generated. Describe any experiments used to assess the effect of a mutation and, where applicable, how potential secondary effects (e.g. second site T-DNA insertions, mosiacism, off-target gene editing) were examined.*

Flow Cytometry

Plots

Confirm that:

☒ The axis labels state the marker and fluorochrome used (e.g. CD4-FITC).

☒ The axis scales are clearly visible. Include numbers along axes only for bottom left plot of group (a 'group' is an analysis of identical markers).

☒ All plots are contour plots with outliers or pseudocolor plots.

☒ A numerical value for number of cells or percentage (with statistics) is provided.

Methodology

Sample preparation	Mouse samples:<br><br>At termination, aorta, blood, plasma, spleen, iliac aortic-draining lymph nodes, and hearts were collected. Blood was collected via cardiac puncture with EDTA-coated syringes (0.5M EDTA, eBioscience). Red blood cells were removed from blood and spleen samples with Ammonium-Chloride-Potassium (ACK) lysis buffer (ThermoFisher). Whole aortas were perfused during harvest with PBS and digested by cutting into small pieces and incubating in digestion mix (450 U/mL collagenase I, 125 U/mL collagenase XI, 60 U/mL DNAse I, 60 U/mL hyaluronidase I, 20 mM HEPES buffer) for 1h at 37°C, shaking at 300 RPM. Tumor infiltrating T cells were isolated by cutting the tumor into small pieces and incubating for 1h at 37°C in a digestion mix (Collagenase IV 1mg/mL, 30 U/mL DNAse I, 60 U/mL Hyaluronidase I and 20mM HEPES buffer). ACK lysis buffer (ThermoFisher) was used to remove red blood cells. Isolated cells were stained with Zombie Aqua (Biolegend) for live/dead exclusion and incubated with extracellular antibody cocktail. Co-production of T-cell cytokines was tested by stimulating splenocytes and pooled aortic digests (n=3-4/pool) with phorbol myristate acetate (PMA)/ionomycin with Brefeldin A (Cell Stimulation Cocktail with Brefeldin A, BioLegend) for 4h at 37°C in media (DMEM supplemented with 10% fetal bovine serum [Gibco]). Staining for IL-2, TNF-α, IFN-γ, Tox, and Ki67 was performed after fixing cells in 2% methanol-free formaldehyde (Thermofisher) and permeabilizing cells with pre-made permeabilization buffer (FoxP3/Transcription Factor Staining Buffer Set, eBioscience).<br><br>Human PBMC PD1 phenotyping:<br><br>PBMCs were isolated from blood samples collected individuals participating in the FIMCOD substudy and kept at -80C. Frozen PBMCs were thawed and cultured in complete RPMI (cRPMI; 10% fetal bovine serum, penicillin/streptomycin, L-Glutamine, sodium pyruvate, non-essential amino acids) in the presence of anti-CD49d/anti-CD28 (BD Biosciences, Cat# 347690) for 24 hours. Cells were washed, stained with Zombie Aqua (Biolegend), and incubated with extracellular antibody cocktail.<br><br>Human PBMC in vitro stimulation:<br><br>PBMCs were thawed and resuspended in cRPMI media. Cells were split into three conditions: cRPMI alone, TCR stimulation and cytokine stimulation. For TCR stimulation, cells were transferred to anti-CD3 (2 µg/ml, clone OKT3) coated wells and supplemented with anti-CD49d/anti-CD28 costimulation (BD Biosciences). For cytokine stimulation, cells were transferred to uncoated wells and 50 ng/ml IL-12p70 (PeproTech, cat# 200-12H-2UG), 50 ng/ml IL-15 (PeproTech, cat# 200-15-2UG) and 250 ng/ml IL-18 (R&D systems, cat# 9124-IL-010, with carrier). Cells were stimulated for a total of 24h and Brefeldin A was added to all wells 5 hours before cells were harvested. Cells were washed, stained with Zombie Aqua (Biolegend) and extracellular antibodies, followed by cell fixation and permeabilization (FIX & PERM Cell Permeabilization Kit, Invitrogen), and intracellular antibody staining for IFN-γ (IFN-γ-APC).
Instrument	Gallios (Beckman Coulter), CytoFLEX (Beckman Coulter), and LSR II (BD Bioscience) flow cytometers were utilized.
Software	Flow cytometry data was analyzed with FlowJo software v10.8.0 (Tree Star Inc., OR, USA).
Cell population abundance	Cell sorting was not performed.
Gating strategy	The main gating strategy for identifying murine PD1 T cell population is outlined in Extended Data Figure 2C and with human PBMCs in Extended Data Figure 7B.

☒ Tick this box to confirm that a figure exemplifying the gating strategy is provided in the Supplementary Information.
